# Characterization of microRNA expression in B cells derived from Japanese black cattle naturally infected with bovine leukemia virus by deep sequencing

**DOI:** 10.1371/journal.pone.0256588

**Published:** 2021-09-10

**Authors:** Chihiro Ochiai, Sonoko Miyauchi, Yuta Kudo, Yuta Naruke, Syuji Yoneyama, Keisuke Tomita, Leng Dongze, Yusuke Chiba, To-ichi Hirata, Toshihiro Ichijo, Kazuya Nagai, Sota Kobayashi, Shinji Yamada, Hirokazu Hikono, Kenji Murakami

**Affiliations:** 1 Cooperative Department of Veterinary Medicine, Faculty of Agriculture, Iwate University, Morioka, Iwate, Japan; 2 Graduate School of Veterinary Sciences, Iwate University, Morioka, Iwate, Japan; 3 Field Science Center, Faculty of Agriculture, Iwate University, Shizukuishi, Iwate, Japan; 4 Division of Bacterial and Parasitic Disease, National Institute of Animal Health, Tsukuba, Ibaraki, Japan; 5 Department of Animal Sciences, Teikyo University of Science, Adachi, Tokyo, Japan; University of Edinburgh, UNITED KINGDOM

## Abstract

Bovine leukemia virus (BLV) is the causative agent of enzootic bovine leukosis (EBL), a malignant B cell lymphoma. However, the mechanisms of BLV-associated lymphomagenesis remain poorly understood. Here, after deep sequencing, we performed comparative analyses of B cell microRNAs (miRNAs) in cattle infected with BLV and those without BLV. In BLV-infected cattle, BLV-derived miRNAs (blv-miRNAs) accounted for 38% of all miRNAs in B cells. Four of these blv-miRNAs (blv-miR-B1-5p, blv-miR-B2-5p, blv-miR-B4-3p, and blv-miR-B5-5p) had highly significant positive correlations with BLV proviral load (PVL). The read counts of 90 host-derived miRNAs (bta-miRNAs) were significantly down-regulated in BLV-infected cattle compared to those in uninfected cattle. Only bta-miR-375 had a positive correlation with PVL in BLV-infected cattle and was highly expressed in the B cell lymphoma tissue of EBL cattle. There were a few bta-miRNAs that correlated with BLV *tax/rex* gene expression; however, BLV *AS1* expression had a significant negative correlation with many of the down-regulated bta-miRNAs that are important for tumor development and/or tumor suppression. These results suggest that BLV promotes lymphomagenesis via *AS1* and blv-miRNAs, rather than *tax/rex*, by down-regulating the expression of bta-miRNAs that have a tumor-suppressing function, and this downregulation is linked to increased PVL.

## Introduction

Bovine leukemia virus (BLV) is an RNA virus belonging to the genus *Delta retrovirus*, family *Retroviridae*, and is closely related to human T-lymphotropic virus-1 (HTLV-1) [1]. BLV is the causative agent of enzootic bovine leukosis (EBL), a malignant B cell lymphoma [2, 3]. Although the welfare consequences may vary according to the location of lymphomas and magnitude of organ involvement, animals suffer when lymphomas have progressed beyond early stages. BLV infection is prevalent worldwide, causing large economic losses in the cattle industry. In Japan, a nationwide survey (2010–2011) of BLV revealed that the prevalence was 28.7% and 40.7% in beef breeding and dairy cattle, respectively [4]. Major dairy producing countries, including the United States, Canada, Argentina, and China, have also reported BLV prevalences of 30% to 50% in their dairy herds [5–8]. The following countries and regions around the world have also reported moderate increases in BLV, with prevalences of 2.3% in Turkey [9], 41.3% in Iran [10], 3.9% in Mongolia [11], 9.7% in the Philippines [12], 21.5% in Egypt [13], 12.6% in South Africa [14], and 62% in Colombia [15]. In 1998, the annual number of EBL outbreaks was reported to be only 99, but by 2019 this had increased to 4,113 [16]. EBL is designated as a notifiable disease by the Act on Domestic Animal Infectious Diseases Control, and any whole carcass that is found to have EBL, upon meat inspection, must be completely discarded. As a result, BLV infection has severely damaged the Japanese beef industry, which is well known for its production of highly expensive Wagyu [17].

The mechanisms by which BLV causes malignant B cell lymphoma remain unclear. Most BLV-infected cows are asymptomatic carriers, with approximately 30% of these developing persistent lymphocytosis (PL) and only 0.1% to 5% developing EBL [2, 3, 18]. The BLV genome uses its own integrase to integrate into the host genome, where it becomes a provirus and persists throughout the life of the host. Several studies have provided evidence that the progression of EBL occurs through the dysregulation of various cellular signaling pathways and is induced by the integration of the BLV genome into the host and the expression of genes that encode proteins, such as Tax, BLV mRNAs, antisense RNAs, and microRNAs (miRNAs) [19–22].

MiRNAs are a large class of small non-coding single-stranded RNAs, 19–25 nucleotides in length, that regulate gene expression at both transcriptional and post-transcriptional levels. MiRNAs bind to complementary sites on the 3’ untranslated region (UTR) of target genes and, consequently, regulate post-transcriptional gene expression via mRNA degradation and translational repression [23]. By targeting multiple transcripts, miRNAs are involved in biological processes such as cell differentiation, proliferation, and apoptosis [24]. It has been reported that miRNAs derived from viruses and their hosts are involved in tumorigenesis [25]. For example in Kaposi sarcoma-associated herpesvirus infection, miRNAs that are derived from the virus participate in the inhibition of apoptosis by the virus and are thus likely to be involved in tumorigenesis [26].

Recently, it has been reported that BLV encodes a conserved cluster of miRNAs that are transcribed by RNA polymerase III (Pol III) [19, 22]. Unlike most host miRNAs, these miRNAs are not processed by the endonuclease Drosha, which allows the viral RNA polymerase II (Pol II) genomic and mRNA transcripts to escape cleavage. Kincaid *et al*. [19], reported that one particular BLV miRNA (miR), blv-miR-B4, has nucleotide sequences that are partially identical to and share common targets with the host miRNA miR-29, which is considered to be associated with tumorigenesis in humans. In an experimental ovine model, BLV miRNAs have been shown to represent approximately 40% of all miRNAs present in the B cells of asymptomatic animals and those in the lymphoma stages of BLV infection [22]. However, it is unclear how miRNAs derived from BLV contribute to the development of EBL.

In this study, we performed comparative analyses of B cell miRNA expression in cattle uninfected and naturally infected with BLV. In these cattle, the relationships between the miRNA expression and BLV proviral load, *tax/rex* gene expression, and *AS1* gene expression were investigated.

## Materials and methods

### Blood and tissue sample collection, serum isolation, and DNA/RNA extraction

Blood was collected from the jugular vein of 16 BLV-naturally infected and 6 BLV-uninfected Japanese Black cattle, bred at the Iwate University Field Science Center. The BLV provirus genomes of all cattle were examined by quantitative PCR (qPCR), as described below, and ELISA using anti-BLV antibodies according to the manufacturer’s instructions (JNC Inc., Tokyo, Japan). Lymphoma tissues were also obtained from five cattle diagnosed with EBL at the Iwate University Field Science Center. Details of animals used in this study are shown in Table 1. All procedures and animals used in this study were approved by the Iwate University Animal Care and Use Committee (no. A201704).

**Table 1:** Animals used in this study.

Animal No.	Breed ^a^	Sex ^b^	Age (Months)	BLV ^c^	EBL ^c^
B0.31	JB	F	14	−	−
B5.31	JB	F	68	−	−
B9.24	JB	F	26	−	−
B9.27	JB	F	23	−	−
B5.23	JB	F	67	−	−
B0.32	JB	F	13	−	−
7546	JB	F	120	+	−
4374	JB	F	33	+	−
8858	JB	C	15	+	−
7566	JB	C	24	+	−
2581	JB	F	131	+	−
2984	JB	C	11	+	−
2985	JB	C	11	+	−
4180	JB	F	84	+	−
2377	JB	F	179	+	−
B8.20	JB	F	40	+	−
B8.40	JB	F	33	+	−
B6.6	JB	F	65	+	−
8170	JB	F	50	+	−
8381	JB	F	12	+	−
1827	JB	F	24	+	−
2983	JB	F	22	+	−
E0425	JB	F	212	+	+
J14	JB	F	78	+	+
J19	JB	F	43	+	+
Iw190523	JB	F	31	+	+
Iw190607	JB	F	33	+	+

^a^ JB, Japanese Black.

^b^ F, female; C, castrate.

^c^ BLV, bovine leukemia virus; EBL, enzootic bovine leukosis; +, positive;–, negative.

Genomic DNA was extracted from EDTA-treated whole blood with a magLEAD^®^ 12gC instrument (Precision System Science, Chiba, Japan) immediately after the blood collection. RNA was extracted from whole blood collected in PAXgene Blood RNA tubes (PreAnalytix, Hombrechtikon, Switzerland) and stored at -70°C for several months after the blood collection. RNA was also extracted from the bovine B cell leukemia cell line KU-17 [27] with TRIzol Reagent (Invitrogen, Carlsbad, CA, USA). These DNA and RNA extraction procedures were performed according to manufacturer’s instructions. Extracted DNA and RNA were stored at -20°C and -70°C, respectively, until analyzed.

### Isolation of B cells from peripheral blood mononuclear cells

EDTA-treated whole blood was layered over 60% percoll (GE Healthcare, Tokyo, Japan) in Leucosep tubes (Greiner Bio-One, Kremsmunster, Austria) and the peripheral blood mononuclear cells (PBMCs) were isolated via density gradient centrifugation for 20 min at 1,000 *g*. The isolated cells (10^8^ cells) were incubated with 1,000 μL of anti-bovine IgM mouse monoclonal antibody (diluted 1:100) (BIG73A; VMRD, Pullman, WA, USA), diluted with MACS buffer [2 mM EDTA, 0.5% BSA in PBS (pH 7.2)], at 4°C for 15 min. The cells were then incubated with anti-mouse IgG microbeads (Miltenyi Biotec, Bergisch Gladbach, Gemany) at 4°C for 15 min. The cells were passed through a cell strainer (EASY strainer; pore size 40 μm, Greiner Bio-One) and applied to a MACS LS column (Miltenyi Biotec) in the magnetic field of a MACS separator (Miltenyi Biotec). After washing three times with MACs buffer, the column was removed from the MACS separator and the magnetically labeled cells were flushed into a collection tube. Approximately 3 x 10^7^–7 x 10^7^ PBMCs were recovered.

The MACS sorted PBMCs (10^6^ cells) were incubated with 20 μL of anti-bovine IgM mouse monoclonal antibody (diluted 1:100) (PIG45A2; VMRD) at 4°C for 15 min. The cells were then stained with 20 μL of FITC-conjugated anti-mouse IgG+IgM antibody (diluted 1:1,000) (#115-096-068; Jackson ImmunoResearch Laboratories, Inc., West Grove, PA, USA) at 4°C for 15 min. After washing twice with PBS, the cells were fixed with 1% paraformaldehyde/PBS. The percentage of IgM^+^ B cells was analyzed on a flow cytometer (Bay Bioscience, Kobe, Japan). FlowJo software (Becton, Dickinson and Company, Franklin Lakes, NJ, USA) was used for flow cytometric data analysis.

### MicroRNA library preparation

Total RNA, containing miRNA, was extracted from B cells (10^7^ cells per animal) and lymphoma tissues using miRNeasy Mini Kits (Qiagen K.K., Tokyo, Japan). The RNA integrity number (RIN) was determined on an Agilent RNA 6000 Nano Bioanalyzer (Agilent Technologies, Santa Clara, CA, USA).

The 3’ and 5’ adaptors were ligated to the total RNA extracted from the isolated B cells with TruSeq Small RNA Library Preparation Kits (Illumina, San Diego, CA, USA). For 3’ adaptor ligation, total RNA was incubated at 70°C for 2 min and then transferred to ice. Subsequently, the following reagents were added to the mixture: 5 μL of 1 μg total RNA, 1 μL of RNA 3’ adaptor, 2 μL of Ligation Buffer, 1 μL of RNase Inhibitor, 1 μL of 10× T4 RNA Ligase 2, Deletion Mutant (Epicentre, Madison, WI, USA). The reaction was incubated at 28°C for 60 min, 1 μL of stop solution (Stop oligo) was added, and the reaction mixture was then incubated at 28°C for 15 min. For 5’ adaptor ligation, a 5’ RNA adaptor was denatured by heating at 70°C for 2 min and was then transferred on ice. The following reagents were added to the 3’ adaptor ligation mixture: 1 μL of RNA 5’ adaptor, 1 μL of ATP (10 mM), 1 μL of T4 RNA ligase, and 11 μL of 3’ adaptor ligation mixture. The reaction mixture was incubated at 28°C for 60 min and then transferred on ice. The sequences of the RNA 3’ adaptor, 5’ adaptor, and Stop oligo are shown in Table 2.

**Table 2:** Primers and probes for library preparation and quantitation of BLV provirus, mRNA, and miRNA expression.

Primer	Sequences (5’–3’)
**For library preparation**	
RNA 5’ adapter	GUUCAGAGUUCUACAGUCCGACGAUC
RNA 3’ adapter (RA3)	TGGAATTCTCGGGTGCCAAGG
Stop solution (Stop Oligo)	GAAUUCCACCACGUUCCCGUGG
RNA_RT	AATGATACGGCGACCACCGAGATCTACACGTTCAGAGTTCTACAGTCCGA
RNA_PCR	AATGATACGGCGACCACCGAGATCTACACGTTCAGAGTTCTACAGTCCGA
RNA_PCR-Index	CAAGCAGAAGACGGCATACGAGAT [Index primer] GTGACTGGAGTTCCTTGGCACCCGAGAATTCCA
Index 1	CGTGAT
Index 2	ACATCG
Index 3	GCCTAA
Index 4	TGGTCA
Index 5	CACTGT
Index 6	ATTGGC
Index 7	GATCTG
Index 8	TCAAGT
Index 9	CTGATC
Index 10	AAGCTA
Index 11	GTAGCC
Index 12	TACAAG
Index 13	TTGACT
Index 14	GGAACT
Index 15	TGACAT
Index 16	GGACGG
Index 17	CTCTAC
Index 18	GCGGAC
Index 19	TTTCAC
Index 20	GGCCAC
Index 21	CGAAAC
**For BLV provirus**	
BLVCG-tax 8008F	CCATGTGACCGGTTACACGTAT
BLVCG-tax 8093R	ACCAATTCGGACCAGGTTAGC
BOS RPPH1-29F	CTACGAGCTGAGTGCGCTTAGTC
BPS RPPH1-97R	CCTATGGCCCTAGTCTCAGACCTT
BLVCG-tax-8034T-probe	FAM-CAGTCCTCAGGCCTT-MGB
BOS RPPH1-54-T-probe	VIC-TCTGTCCATTGTCCC-MGB
**For mRNA and miRNA expression**	
BLV_tax/rex_mRNA_F	CAGATGGCAAGTGTTGTTGGTT
BLV_tax/rex_mRNA_R	GATGGTGACATCATTGGACAAAA
BLV_AS1 real_F	ATTTTATTAATTTATCAGCAGGTAATG
BLV_AS1 real_R1	AGTGCCCATAAAGTCCCTTC
boGAPDH_F	CCCAGAATATCATCCCTGCTT
boGAPDH_R	GCAGGTCAGATCCACAACAGA
boHBP1rt-F	TTCAACTGCTTGGCACTGTTTT
boHBP1rt-R	CCATTCCTTATTGCTTCCCTTATG
boACTBrt-F	AACCAGTTCGCCATGGATGA
boACTBrt-R	AAGCCGGCCTTGCACAT
bta-miR-375-F	TTTTGTTCGTTCGGCTCG
bta-miR-16a-F	TAGCAGCACGTAAATATTGGTG

### Reverse transcription of adapter ligation products

RNA RT Primer (1 μL) was added to 6 μL of the adaptor ligation mixture, described in the previous section, heated at 70°C for 2 min, and then immediately placed on ice. The following reagents were then added to the ligation mixture: 2 μL of 5× First Strand Buffer, 0.5 μL of 12.5 mM dNTP mix, 1 μL of 100 mM DTT, 1 μL of RNase Inhibitor, and 1 μL of SuperScript II Reverse Transcriptase (Thermo Fisher Scientific, Waltham, MA, USA). The reaction mixture was then incubated at 50°C for 60 min. The sequence of the RNA RT Primer is shown in Table 2.

### PCR amplification and purification of PCR products

The following reagents were added to 12.5 μL of reverse transcription reaction mixture described in the previous section: 25 μL of PCR mix, 2 μL of miRNA PCR primer, 2 μL of miRNA PCR primer Index, and 8.5 μL of nuclease-free purified water to make the total reaction mixture up to 50 μL. The PCRs were performed under the following conditions: initial denaturation at 98°C for 30 s; followed by 15 cycles of heat denaturation at 98°C for 10 s, annealing at 60°C for 30 s, and extension at 72°C for 15 s; then a final extension at 72°C for 1 min. The sequences of the primers used are shown in Table 2. The PCR products (145 bp to 160 bp) were purified on 6% Novex TBE gels (Life Technologies, Waltham, MA, USA), following the manufacturer’s instructions. The PCR products were evaluated with a microchip based capillary electrophoresis system (MultiNA, Shimadzu, Tokyo, Japan), and the concentrations were measured on a Qubit fluorometer (Thermo Fisher Scientific).

### MicroRNA deep sequencing and analysis

MicroRNA analysis was performed using a MiniSeq Sequencing System (Ilumina). The libraries were diluted to 1 nM with 10 mM Tris HCl (pH 8.5) and made up to 5 μL each, to which, 5 μL of 2-fold diluted sodium hydroxide solution (Fluka Analytical, St. Gallen, Switzerland) was added. The mixture was incubated for 5 min at room temperature, and then 5 μL of 200 mM Tris HCl (pH 7) was added and the reaction mixture kept on ice. The mixed library reaction was diluted to 1.8 pM with hybridization buffer (Ilumina), and then 500 μL of the reaction mixture was applied to a MiniSeq High Output Reagent Cartridge (Ilumina). Deep sequence analysis was performed according to the manufacturer’s recommended protocol for small RNA sequencing. Afterwards, sequencing reads were processed with CLC Genomics Workbench software (Ver. 9.5.5; Qiagen KK) to obtain the final miRNA counts for each sample (see Qiagen tutorial manual for small RNA Analysis using Illumina Data for detail; https://resources.qiagenbioinformatics.com/tutorials/Small_RNA_analysis_Illumina.pdf). Briefly, adapter sequences were removed from the partial adapter sequences of the FASTQ file. The adapter trimming parameters were set to default values; i.e., mismatch cost and gap cost were 2 and 3, respectively; match threshold was selected to “allow end matches”; and the minimum score at the end was set to 6. Subsequently, for sequence filtering, the minimum and maximum length values were used as default values; i.e., reads below length of 15 and above length of 55 were discarded, and the sample threshold for the minimum sampling count was set to 1. The number of copies of each of the resulting small RNAs was counted. To annotate the small RNA sample, the bovine miRNA database in miRBase 22 [28] (http://www.mirbase.org/blog/2018/03/mirbase-22-release/) was downloaded. The trimmed sequences were compared against the bovine miRNA database with CLC software for miRNA gene identification, annotation, and quantification. Specified match parameters were set to default values: mature length variants (IsomiRs) were set to additional upstream bases, 2; additional downstream bases, 2; missing upstream bases, 2; and missing downstream bases, 2. The alignment setting was set to a maximum of 2 mismatches.

### Quantification of BLV provirus

We performed duplex quantitative PCR (qPCR) that targeted the BLV *tax/rex* gene region, and bovine RPPH1 gene as an internal control. The qPCR was performed under the following conditions: initial denaturation at 95°C for 20 s, followed by 40 amplification cycles of denaturation at 95ºC for 1 s and annealing/extension at 60ºC for 20 s. Reaction mixtures consisted of 5 μL of template genomic DNA derived from whole blood, 10 μL of Premix Ex Taq (Probe qPCR; Takara Bio, Shiga, Japan), 0.4 μL each of 10 μM *tax/rex* forward and reverse primers, 0.3 μL each of 10 μM RPPH1 Forward and reverse primers, 0.8 μL of 2.5 μM FITC-labeled TaqMan MGB *tax/rex* probe (Life Technologies, Tokyo, Japan), 0.8 μL of 2.5 μM VIC-labeled TaqMan MGB RPPH1 probe (Life Technologies), 0.4 μL of ROX Reference Dye (Takara Bio), and deionized water to make the total rection volume up to 20 μL. The primer sequences used in this study are shown in Table 2. The qPCRs were performed on a QuantStudio^™^ 3 Real-Time PCR System (Applied Biosystems, Life Technologies, Foster City, CA, USA). Standard curves were generated by creating 10-fold serial dilutions of standard plasmids that contained the relevant BLV *tax/rex* or bovine RPPH1 genes, amplified by the appropriate PCR primers. The standards for calibration ranged from 10^0^ to 10^5^ copies/reaction and were run in duplicate. The number of BLV copies was indicated as proviral load per 10 ng DNA. The percent of BLV-infected cells was calculated by the following equation (as there were two copies of the RPPH1 gene per cell):

[%ofBLV-infectedcells=BLVtax/rexcopynumber÷(RPPH1copynumber÷2)×100].

### Quantification of mRNA and miRNA expression by quantitative RT-PCR (qRT-PCR)

SYBR Prime Script RT-PCR Kits (Takara Bio) were used for *tax/rex*, *AS1*, and bovine HMG box-containing protein 1 (*HBP1*) mRNA and miScript II RT Kits (Qiagen KK) were used for bta-miR-375. For *tax/rex*, *AS1*, and bovine *HBP1* mRNA, reverse transcription reaction mixtures consisted of 400 ng/μL of template RNA, derived from whole blood; 4 μL of 5× primeScript Buffer; 1 μL of 50 μM Oligo-dT primer; 1 μL of 100 μM random 6-mer primer; 1 μL of PrimeScript RT Enzyme Mix 1; and RNase-free water to make the total reaction volume up to 10 μL. The reaction was incubated at 37°C for 15 min, and then heated at 85°C for 5 s for enzyme inactivation and placed on ice. For bta-miR-375, reverse transcription reaction mixtures consisted of 300 ng of template RNA, derived from isolated B cells and lymphoma tissues; 4 μL of 5x miScript HiFlex Buffer; 2 μL of 10x miScript Nucleic Mix; 2 μL of miScript Reverse Transcriptase Mix, and RNase-free water up to a total reaction volume of 20 μL. The reaction mixture was incubated at 37°C for 60 min, and then heated at 95°C for 5 min for enzyme inactivation and placed on ice. The concentrations of cDNAs obtained were calculated by absorbance at 260 nm on a NanoDrop One (Thermo Fisher Scientific K.K).

Quantitative RT-PCRs that targeted *tax/rex* and *AS1* mRNAs were performed with *GAPDH* mRNA as the internal control. Bovine *HBP1* mRNA was targeted with beta actin (*ACTB*) mRNA as an internal control [29], and bta-miR-375 was targeted with bta-miR-16a-5p as an internal control. For *tax/rex*, *AS1*, and bovine *HBP1* mRNA, reaction mixtures consisted of 40 ng/5 μL of cDNA, 0.8 μL each of 10 μM forward and reverse primers, 10 μL of SYBR Premix Ex Taq (Takara Bio), 0.4 μL of Rox Reference Dye (Takara Bio), and 3 μL of sterilized ultrapure water. PCRs were performed under the following conditions: initial denaturation at 95°C for 30 s, 40 cycles of denaturation at 95°C for 5 s and annealing/extension at 60°C for 30 s. The gene copy number was calculated via the standard curve method. For bta-miR-375, miScript SYBR green PCR Kits (Qiagen KK) were used. Reaction mixtures consisted of 3 ng of cDNA, 2.5 μL of 10x miScript Universal Primer, 2.5 μL of microRNA-specific primer, 12.5 μL of 2x QuantiTect SYBR Green PCR Master Mix, and sterilized ultrapure water up to a total reaction volume of 25 μL. PCRs were performed under the following conditions: initial denaturation at 95°C for 15 min; 40 cycles of denaturation at 94°C for 15 s, annealing at 55°C for 30 s, and extension at 70°C for 30 s. The relative miRNA expression levels were calculated using the ΔΔCT comparative method by Quantstudio^™^ design and analysis software (Version 2.4, Thermo Fisher Scientific). The sequences of the primers used are shown in Table 2.

### Statistical analysis

Differences in expression of BLV miRNAs (blv-miRNAs) and bovine miRNAs (bta-miRNAs) between BLV-infected and BLV-uninfected cattle were assessed using Mann-Whitney test. Correlations between parameters in BLV-infected cattle were assessed by the Spearman’s correlation coefficients. Differences in bta-miR-375 expression among BLV negative, BLV-positive, and EBL cattle were assessed by Kruskal-Wallis test with Steel- Dwass post-hoc test. Differences in expression of BLV miRNAs (blv-miRNAs) and bovine miRNAs (bta-miRNAs) between BLV *AS1* high expression cattle and low expression cattle were assessed using a Mann-Whitney test. These data analyses were performed by R, a language and environment for statistical computing (R Core Team, 2020. URL https://www.R-project.org/). Statistical significance was determined as *p* < 0.05.

## Results

### MicroRNA sequencing reads in B cells of BLV-infected and uninfected cattle

B cells were isolated from 16 BLV-infected and 6 uninfected cattle at purity levels between 82% and 97%. The RINs of RNA samples derived from B cells were 6.9 to 10. The numbers of miRNAs that were read in these RNA samples were between 1.33 × 10^6^ and 4.12 × 10^6^. Among these miRNAs, 614 bovine-derived miRNAs (bta-miRNAs) were detected out of 1,064 currently registered in the database (miRBase) (S1 Table). In addition, the 10 BLV provirus-derived miRNAs (blv-miR), which were previously reported [22], were also detected (S2 Table).

**Fig 1:**
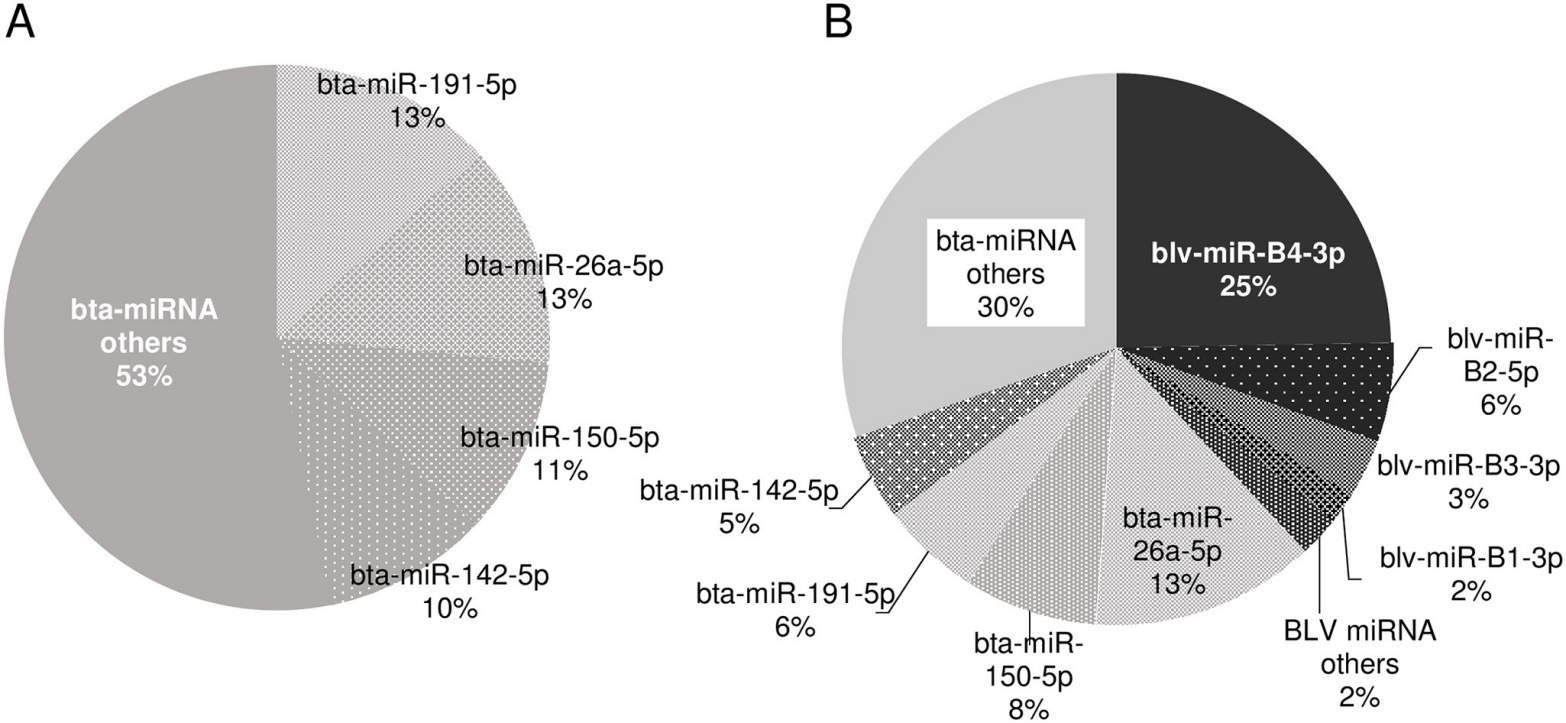
Percentage of miRNAs expressed in B cells derived from BLV-infected cattle and healthy cattle without BLV infection. The average ratios of miRNAs expressed in B cells made up of bovine-derived miRNA (bta-miRNA) and/or BLV-derived miRNA (blv-miRNA) were calculated. (A) Healthy cattle without BLV infection, (B) BLV-infected cattle.

In BLV-uninfected cattle, four bovine-derived miRNAs accounted for 47% of all miRNAs expressed in B cells: bta-miR-191-5p (13%), bta-miR-26a-5p (13%), bta-miR-150-5p (11%), and bta-miR-142-5p (10%). Whereas, in BLV-infected cattle, a BLV provirus-derived miRNA, blv-miR-B4-3p, was highly expressed in B cells (25%) and blv-miRNAs accounted for 38% of all miRNAs expressed in B cells (Fig 1, S2 Table).

The miRNA bta-miR-16a-5p had the most consistent number of copies among all 22 cattle. The read counts of bta-miRNAs, normalized using the bta-miR-16a-5p read count, were compared between BLV-infected and uninfected cattle. We focused on 49 bta-miRNAs because these miRNAs differed significantly between BLV-infected and uninfected cattle. Among the 49 bta-miRNAs, 48 bta-miRNAs in BLV-infected cattle were significantly decreased compared to those in uninfected cattle (*p* < 0.05, Table 3). In particular, four bta-miRNAs (bta-miR-191-5p, bta-miR-423-3p, bta-miR-92b-3p, and bta-miR-361-5p) showed the higher ratios (> 5.0) of read counts between BLV-infected and -negative cattle, and none of the bta-miRNA interquartile ranges overlapped. Only bta-miR-375-3p expression in BLV-infected cattle was significantly increased compared with those in uninfected cattle (*p* = 0.0061).

**Table 3:** Comparison of bovine miRNA (bta-miRNAs) sequencing reads between BLV positive and BLV negative cattle.

Name of miRNA	BLV-infected (n = 16)		BLV-negative (n = 6)		*p* value ^c^	Oncogene (ONC) or tumor suppressor (TS)	Reference
	Median ^a^	IQR ^b^	Median ^a^	IQR ^b^			
bta-miR-191-5p	22,468	5,223, 103,053	172,846	151,559, 233,390	0.0002	TS/ONC	[30, 31]
bta-miR-26a-5p	109,376	51,793, 127,643	152,887	138,543, 174,790	0.0034	TS/ONC	[32–34]
bta-miR-142-5p	32,660	9,553, 72,034	126,224	113,628, 162,294	0.0034	TS	[35]
bta-miR-150-5p	59,170	35,803, 63,672	105,905	101,347, 109,417	0.0003	TS/ONC	[36, 37]
bta-miR-22-3p	15,139	8,311, 34,125	48,605	33,982, 56,215	0.0045	TS	[38]
bta-miR-26b-5p	17,967	11,520, 21,192	25,071	22,133, 28,963	0.0133	TS	[32, 39, 40]
bta-miR-375-3p	52,366	31,278, 65,148	18,518	15,515, 19,965	0.0061	TS/ONC	[32, 41]
bta-miR-186-5p	5,541	1,793, 15,309	26,465	23,319, 29,157	0.0017	TS	[32, 42]
bta-miR-16b-5p	10,556	9,974, 12,339	15,032	13,644, 15,920	0.0266	TS	[32, 43]
bta-miR-30c-5p	7,413	4,527, 9,226	13,803	10,992, 15,521	0.0133	TS	[44]
bta-miR-29a-3p	5,052	3,053, 6,517	12,687	10,830, 15,162	0.0005	TS/ONC	[32, 45, 46]
bta-miR-192-5p	6,767	6,042, 9,642	11,939	8,521, 14,829	0.0487	TS/ONC	[47]
bta-miR-151-5p	2,204	1,159, 5,938	8,774	8,136, 9,243	0.0034	TS	[48]
bta-miR-6119-5p	3,487	883, 6,666	7,446	6,189, 8,983	0.0328	Other	[49]
bta-miR-342-3p	1,611	641, 2,689	5,769	4,894, 7,126	0.0045	TS	[50, 51]
bta-miR-425-5p	3,162	1,867, 4,587	6,565	6,395, 6,883	0.0005	TS/ONC	[52, 53]
bta-miR-423-3p	795	205, 3,900	5,678	3,994, 9,333	0.0328	TS	[54]
bta-miR-146a-5p	1,747	831, 3,229	4,626	3,783, 7,467	0.0170	TS/ONC	[55, 56]
bta-miR-142-3p	2,446	1,478, 2,860	4,786	3,747, 5,145	0.0008	TS	[57]
bta-miR-29c-3p	1,705	935, 2,020	4,033	3,347, 4,803	0.0012	TS/ONC	[32, 45, 46]
bta-miR-423-5p	779	443, 2,531	3,608	2,387, 4,326	0.0426	TS/ONC	[58, 59]
bta-miR-151-3p	1,074	842, 2,205	3,258	2,842, 3,642	0.0080	TS	[48]
bta-miR-155-5p	1,647	1,263, 2,248	2,684	2,382, 3,511	0.0328	TS/ONC	[60, 61]
bta-miR-138-5p	1,407	847, 2,026	2,426	1,955, 2,783	0.0402	TS	[62, 63]
bta-miR-148b-3p	1,608	1,110, 1,734	2,585	1,920, 3,148	0.0133	TS	[64]
bta-miR-27a-3p	806	353, 1,483	2,448	1,778, 3,153	0.0034	TS/ONC	[32, 65, 66]
bta-miR-221-3p	531	251, 1,031	1,918	1,537, 2,227	0.0022	TS/ONC	[32, 45]
bta-miR-197-3p	664	461, 848	1,290	1,100, 1,343	0.0017	TS	[67, 68]
bta-let-7d-5p	960	726, 1,235	1,448	1,319, 1,548	0.0165	Other	[69]
bta-miR-484-5p	294	159, 496	1,101	787, 1,145	0.0001	TS/ONC	[70, 71]
bta-miR-92b-3p	260	96, 686	1,292	1,178, 1,558	0.0356	TS/ONC	[72, 73]
bta-miR-361-5p	205	38, 787	1,301	1,002, 1,603	0.0133	TS	[74]
bta-miR-28-5p	433	202, 795	1,111	999, 1,203	0.0017	TS/ONC	[75, 76]
bta-miR-23a-3p	670	269, 936	1,047	955, 1,194	0.0266	TS/ONC	[77, 78]
bta-miR-106b-5p	458	291, 719	1,038	918, 1,123	0.0024	ONC	[79]
bta-miR-2285f-3p	254	77, 558	876	640, 1,218	0.0183	Other	[80]
bta-miR-421-3p	416	283, 635	749	657, 902	0.0170	TS/ONC	[32, 81, 82]
bta-miR-532-5p	308	207, 345	526	465, 839	0.0057	TS/ONC	[83, 84]
bta-miR-363-3p	237	201, 416	619	567, 639	0.0135	TS	[85]
bta-miR-24-2-3p	256	187, 398	517	460, 601	0.0071	ONC	[77]
bta-miR-339b-5p	208	83, 313	510	407, 576	0.0061	Other	[86]
bta-miR-326-3p	242	220, 293	433	397, 480	0.0109	TS	[87]
bta-miR-32-5p	193	150, 250	399	310, 464	0.0005	TS/ONC	[88, 89]
bta-miR-194-5p	175	126, 286	363	360, 427	0.0017	TS	[32, 90]
bta-miR-107-3p	259	146, 314	385	337, 447	0.0213	Other	[91]
bta-miR-874-3p	113	35, 155	258	187, 363	0.0223	TS	[92]
bta-miR-374a-5p	142	66, 193	267	233, 288	0.0165	TS/ONC	[93, 94]
bta-miR-6524-3p	151	129, 177	248	215, 282	0.0034	Other	[95]
bta-miR-1307-3p	114	77, 140	225	183, 233	0.0024	TS/ONC	[96, 97]

^a^ Read counts of bta-miRNAs were normalized to bta-miR-16a-5p (accession No. LC600681) read count (x 10,000).

^b^ Interquartile range.

^c^ Statistically significant p values were calculated by Mann-Whitney test.

The nucleotide sequences of bta-miRNAs and blv-miRNAs obtained and used in this study have been submitted to the DDBJ/EMBL/GenBank DNA databases under the accession numbers LC600590-LC600593, LC600597, LC600602, LC600604, LC600605, LC600608-LC600610, LC600612, LC600615-LC600619, LC600621, LC600623, LC600627, LC600629-LC600631, LC600634, LC600635, LC600637, LC600641, LC600643, LC600644, LC600646-LC600648, LC600650, LC600652, LC600653, LC600658, LC600659, LC600662-LC600664, LC600666-LC600669, LC600671, LC600673, LC600676, LC600677, LC600679, LC600681, and LC600682-LC600691.

### Correlation between miRNA sequencing reads and BLV proviral load in BLV-infected cattle

**Fig 2:**
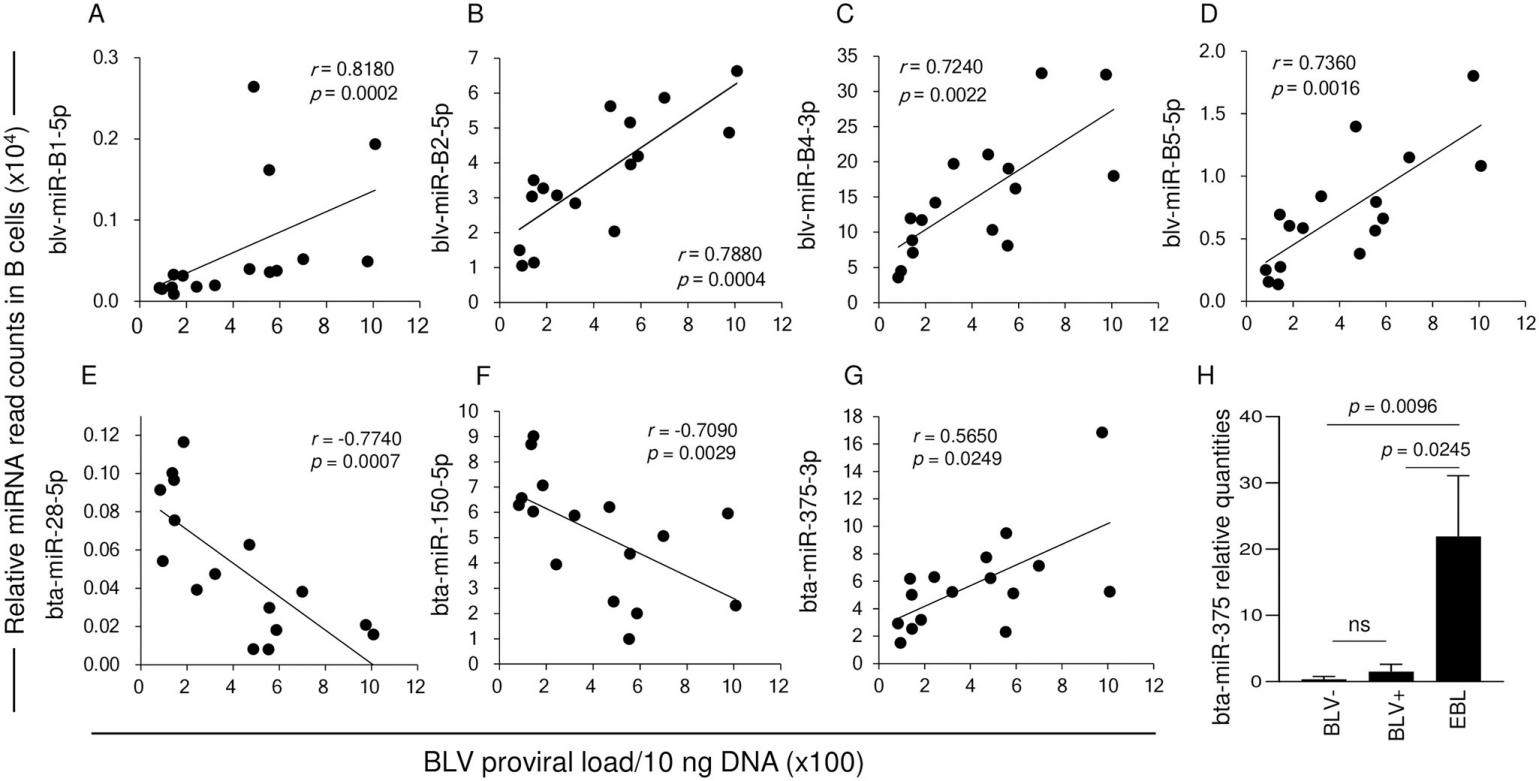
Correlations between BLV proviral load and BLV miRNAs (blv-miRNAs) and bovine-derived miRNAs (bta-miRNAs), and expression levels of bta-miR-375 among BLV-uninfected, BLV-infected, and enzootic bovine leukosis (EBL) cattle. (A–D) Correlations between BLV PVL and blv-miRNA read counts in B cells derived from BLV-infected cattle. (E–G) Correlations between BLV PVL and bta-miRNAs in B cells derived from BLV-infected cattle. All read counts of blv-miRNAs and bta-miRNAs were normalized to the read counts of bta-miR-16a-5p. Data were analyzed by Spearman’s correlation coefficient test; *r*, correlation coefficient; *p*, *p* value. (H) Levels of bta-miR-375 expression, measured by quantitative RT-PCR in B cells derived from BLV-uninfected (n = 8) and BLV-infected (n = 5) cattle, and in B cell lymphomas (n = 5) derived from EBL cattle. Data were analyzed by Kruskal-Wallis test followed by Steel-Dwass post-hoc test.

The read counts of four blv-miRNAs (blv-miR-B1-5p, blv-miR-B2-5p, blv-miR-B4-3p, and blv-miR-B5-5p) had a strong positive correlation with BLV PVL (correlation coefficient (*r*) > 0.7, *p* < 0.05, Fig 2A–2D). Among the 49 bta-miRNAs with read counts that differed significantly between BLV-infected and uninfected cattle, 31 bta-miRNAs negatively correlated with PVL (Table 4). In particular, three bta-miRNAs (bta-miR-28-5p, bta-miR-150-5p, and bta-miR-197-3p) had a strong negative correlation with PVL (*r* < -0.7, *p* <0.05, Fig 2E–2F), followed by 13 bta-miRNAs (bta-miR-221-3p, bta-miR-22-3p, bta-miR-151-5p, bta-miR-484-5p, bta-miR-194-5p, bta-miR-425-5p, bta-miR-151-3p, bta-miR-146a-5p, bta-miR-1307-3p, bta-miR-363-3p, bta-miR-874-3p, bta-miR-106b-5p, and bta-miR-421-3p) with relatively weaker negative correlation coefficients (-0.7 < *r* < -0.6, *p <* 0.05). Only bta-miR-375-3p had a significant positive correlation (*r* = 0.565, *p =* 0.0249) with PVL (Fig 2G, Table 4). When bta-miR-375 expression was compared among BLV-uninfected, BLV-infected, and cattle with EBL via quantitative RT-PCR, the levels were significantly higher in EBL cattle than in BLV-uninfected and BLV-infected cattle (BLV-uninfected vs EBL, *p* = 0.0096; BLV-infected vs EBL, *p* = 0.0245) (Fig 2H).

**Table 4:** Correlation between bta-miRNA sequencing reads and BLV proviral load (PVL) and *AS1* mRNA expression.

Name of miRNA	BLV proviral load (PVL)		*tax/rex* mRNA		*AS1* mRNA		Oncogene (ONC) or tumor suppressor (TS)
	*r* ^a^	*p* value	*r* ^a^	*p* value	*r* ^a^	*p* value	
bta-miR-28-5p	-0.774	0.00068	-0.597	0.0166	-0.626	0.0111	TS/ONC
bta-miR-150-5p	-0.709	0.0029	-0.479	ns	-0.735	0.0170	TS/ONC
bta-miR-197-3p	-0.709	0.0029	-0.562	0.0258	-0.668	0.0060	TS/ONC
bta-miR-221-3p	-0.688	0.00421	-0.54	0.0308	-0.641	0.0090	TS/ONC
bta-miR-22-3p	-0.685	0.00443	-0.521	0.041	-0.550	0.0296	TS
bta-miR-151-5p	-0.685	0.00443	-0.503	0.0493	-0.603	0.0154	TS
bta-miR-484-5p	-0.676	0.00515	-0.409	ns	-0.732	0.0018	TS/ONC
bta-miR-194-5p	-0.676	0.00515	-0.444	ns	-0.532	0.0361	TS
bta-miR-425-5p	-0.662	0.00654	-0.479	ns	-0.632	0.0102	TS/ONC
bta-miR-151-3p	-0.659	0.00685	-0.462	ns	-0.547	0.0306	TS
bta-miR-146a-5p	-0.629	0.0107	-0.368	ns	-0.641	0.0090	TS
bta-miR-1307-3p	-0.626	0.0111	-0.494	ns	-0.709	0.0029	TS/ONC
bta-miR-363-3p	-0.612	0.0136	-0.374	ns	-0.479	ns	TS
bta-miR-874-3p	-0.612	0.0136	-0.497	ns	-0.453	ns	TS
bta-miR-106b-5p	-0.609	0.0142	-0.406	ns	-0.644	0.0086	Other
bta-miR-421-3p	-0.600	0.0160	-0.456	ns	-0.618	0.0126	TS/ONC
bta-miR-142-5p	-0.597	0.0166	-0.429	ns	-0.553	0.0286	TS
bta-miR-2285f-3p	-0.597	0.0166	-0.456	ns	-0.503	0.0493	TS/ONC
bta-miR-186-5p	-0.585	0.0193	-0.426	ns	-0.535	0.0349	TS
bta-miR-24-2-3p	-0.585	0.0193	-0.511	0.0432	-0.518	0.0423	Other
bta-miR-29a-3p	-0.582	0.0200	-0.462	ns	-0.566	0.0240	TS/ONC
bta-miR-342-3p	-0.579	0.0208	-0.388	ns	-0.632	0.0102	TS/ONC
bta-miR-6119-5p	-0.556	0.0276	-0.409	ns	-0.568	0.0240	Other
bta-miR-339b-5p	-0.556	0.0276	-0.4	ns	-0.488	ns	TS
bta-miR-191-5p	-0.550	0.0296	-0.385	ns	-0.609	0.0142	TS
bta-miR-138-5p	-0.55	0.0296	-0.232	ns	-0.541	0.0327	TS
bta-miR-23a-3p	-0.544	0.0316	-0.341	ns	-0.632	0.0102	ONC
bta-miR-423-5p	-0.535	0.0349	-0.366	ns	-0.556	0.0286	TS
bta-miR-27a-3p	-0.524	0.0397	-0.385	ns	-0.544	0.0316	TS/ONC
bta-let-7d-5p	-0.515	0.0437	-0.411	ns	-0.697	0.0036	Other
bta-miR-29c-3p	-0.509	0.0464	-0.429	ns	-0.553	0.0286	TS/ONC
bta-miR-92b-3p	-0.491	ns	-0.302	ns	-0.641	0.0090	TS
bta-miR-423-3p	-0.476	ns	-0.306	ns	-0.624	0.0116	TS/ONC
bta-miR-326-3p	-0.468	ns	-0.457	ns	-0.438	ns	TS/ONC
bta-miR-32-5p	-0.462	ns	-0.318	ns	-0.453	ns	TS
bta-miR-142-3p	-0.438	ns	-0.253	ns	-0.268	ns	TS/ONC
bta-miR-155-5p	-0.432	ns	-0.397	ns	-0.571	0.0232	TS/ONC
bta-miR-30c-5p	-0.415	ns	-0.221	ns	-0.5	ns	TS/ONC
bta-miR-361-5p	-0.412	ns	-0.265	ns	-0.488	ns	TS/ONC
bta-miR-26a-5p	-0.409	ns	-0.279	ns	-0.524	0.0397	TS
bta-miR-148b-3p	-0.397	ns	-0.203	ns	-0.488	ns	TS/ONC
bta-miR-16b-5p	-0.374	ns	-0.121	ns	-0.468	ns	TS
bta-miR-532-5p	-0.356	ns	-0.255	ns	-0.671	0.0057	TS
bta-miR-374a-5p	-0.344	ns	-0.162	ns	-0.412	0.1140	ONC
bta-miR-26b-5p	-0.309	ns	-0.315	ns	-0.265	0.3210	TS/ONC
bta-miR-107-3p	-0.306	ns	-0.25	ns	-0.565	0.0243	Other
bta-miR-192-5p	-0.085	ns	-0.221	ns	-0.229	ns	TS
bta-miR-6524-3p	0.359	ns	0.400	ns	0.288	ns	TS/ONC
bta-miR-375-3p	0.565	0.0249	0.415	ns	0.174	ns	TS

^a^ The correlation coefficients were analyzed by Spearman’s correlation test.

ns, no significant difference.

### Correlation between miRNA copies and BLV *tax/rex* and *AS1* expression in BLV-infected cattle

The expression of BLV *tax/rex* and *AS1* genes in PBMCs were quantified by qRT-PCR. BLV *tax/rex* and *AS1* mRNA copy numbers were correlated against blv-miRNAs and bta-miRNAs copies. BVL-infected cattle had between 1.6 and 91 BLV *tax/rex* mRNA copies per 10^4^ B cells and 2.1 to 1,388 *AS1* mRNA copies per 10^4^ B cells. There was no significant correlation between *tax/rex* and *AS1* mRNA expression (S1A and S1B Fig).

**Fig 3:**
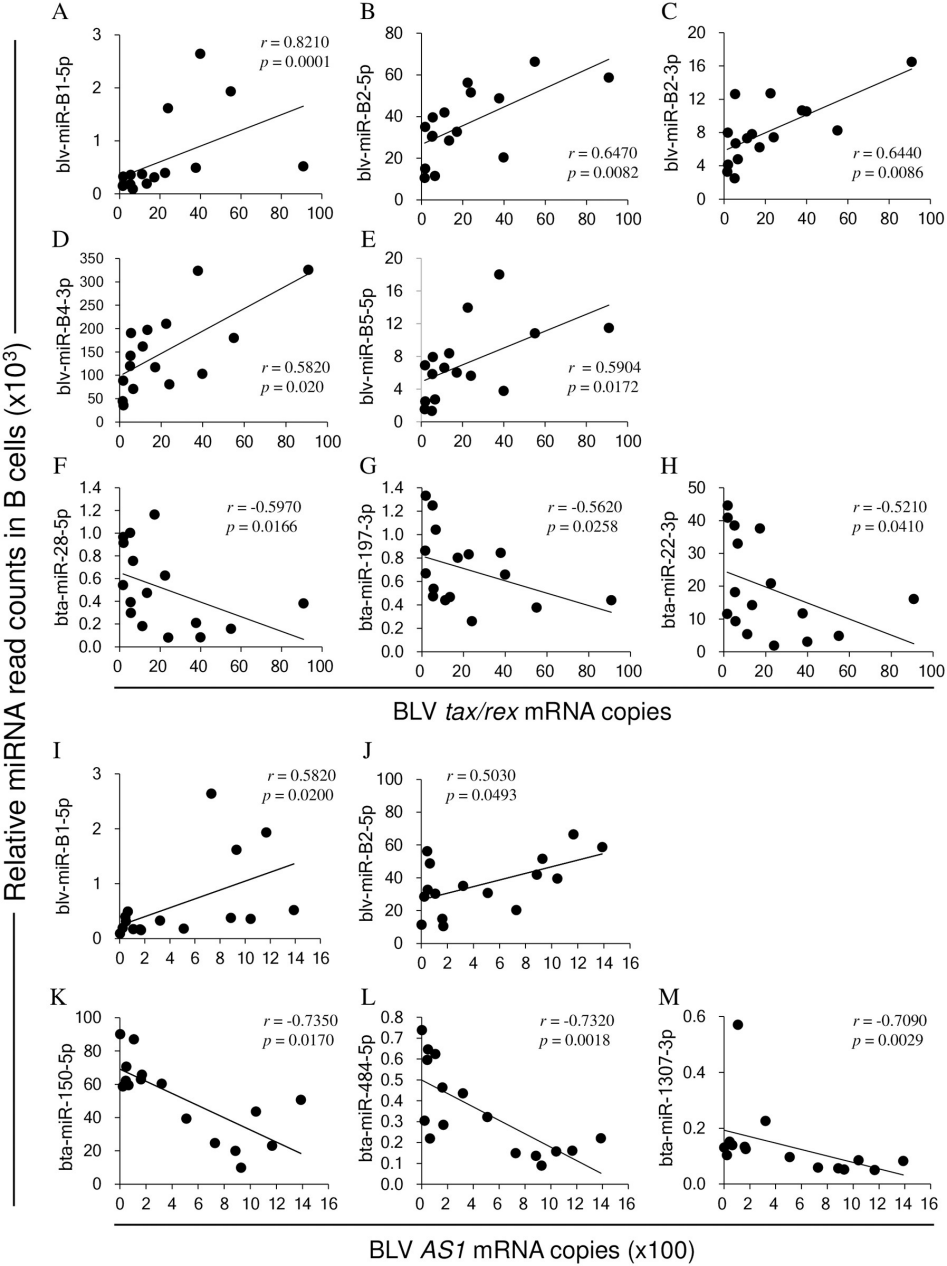
Correlations between BLV *tax/rex* and *AS1* mRNA expression levels against BLV miRNA (blv-miRNA) and bovine-derived miRNA (bta-miRNA) read counts in B cells derived from BLV-infected cattle. All read counts of blv-miRNAs and bta-miRNAs were normalized to read counts of bta-miR-16a-5p. (A–H) BLV *tax/rex* and (I–M) *AS1* mRNA copy numbers were normalized to *GAPDH* mRNA copy number. Data were analyzed by Spearman’s correlation coefficient test; *r*, correlation coefficient; *p*, *p* value.

BLV *tax/rex* mRNA copy number positively correlated with five blv-miRNAs (blv-miR-B1-5p, blv-miR-B2-3p, blv-miR-B2-5p, blv-miR-B4-3p, and blv-miR-B5-5p) (Fig 3A–3E). In addition, BLV *tax*/*rex* mRNA copy number negatively correlated with bta-miR-28-5p (*r* = -0.597, *p* = 0.0166), bta-miR-197-3p (*r* = -0.562, *p* = 0.0258), bta-miR-22-3p (*r* = -0.521, *p* = 0.041), bta-miR-24-2-3p (*r* = -0.511, *p* = 0.0432), and bta-miR-151-5p (*r* = -0.503, *p* = 0.0493) (Fig 3F–3H).

There was a positive correlation between *AS1* mRNA copy number and two blv-miRNAs (blv-miR-B1-5p and blv-miR-B2-5p) (Fig 3I and 3J). Among the 49 bta-miRNAs that had significantly different read counts between BLV-infected and BLV-uninfected cattle, 34 of them had a significant negative correlation with *AS1* mRNA expression (Table 4). In particular, three bta-miRNAs (bta-miR-150-5p, bta-miR-484-5p, and bta-miR-1307-3p) had a strong negative correlation with *AS1* mRNA (*r* < -0.7, *p* <0.05) (Fig 3K–3M), followed by 15 bta-miRNAs (bta-miR-191-5p, bta-miR-151-5p, bta-miR-342-3p, bta-miR-425-5p, bta-miR-423-3p, bta-miR-146a-5p, bta-miR-221-3p, bta-miR-197-3p, bta-let-7d-5p, bta-miR-92b-3p, bta-miR-28-5p, bta-miR-23a-3p, bta-miR-106b-5p, bta-miR-421-3p, and bta-miR-532-5p) that had relatively weaker negative correlation coefficients (*p <* 0.05).

#### HMG-box transcription factor 1 (HBP1) expression

The expression levels of *HBP1* mRNA in B cells derived from BLV-infected cattle did not differ from those of BLV-uninfected cattle. *HBP1* mRNA expression in a bovine B cell tumor cell line, KU-17 was lower than that in B cells derived from both BLV-infected and -uninfected cattle (S2 Fig).

## Discussion

In this study, we performed deep sequencing analysis to comprehensively compare miRNAs expressed in B cells derived from BLV-infected healthy cattle and those derived from BLV-uninfected cattle and determined the correlations between B cell miRNAs and the pathogenesis of BLV. Furthermore, the correlations between B cell miRNAs and BLV proviral load (PVL), BLV *tax/rex* and *AS1* mRNA expression were also investigated.

Ten BLV provirus-derived microRNAs (blv-miRNAs) were detected in B cells derived from BLV-infected cattle, and these blv-miRNAs accounted for 38% of all detected miRNAs. This is in agreement with a study that reported that approximately 40% of total miRNAs were blv-miRNAs in B cell lymphoma derived from sheep experimentally infected with BLV [22]. These results suggest that blv-miRNAs are constantly expressed at a high rate in B cells in healthy BLV-infected cattle, from the asymptomatic stage to the onset of EBL.

The risk of EBL onset in BLV-infected cattle harboring higher PVLs is higher than that in BLV-infected cattle harboring lower PVLs [98]. The read counts of four blv-miRNAs (blv-miR-B1-5p, blv-miR-B2-5p, blv-miR-B4-3p, and blv-miR-B5-5p) had strong positive correlations with PVL. Among these blv-miRNAs, blv-miR-B4-3p had the highest sequencing reads, which is in agreement with a previous study that showed blv-miR-B4-3p had the highest number of read counts in B-cell lymphoma derived from sheep experimentally infected with BLV [22]. The blv-miR-B4-3p has seven bases in common with the 5’ flanking region of the host genome-derived miR-29 family (miR-29a, miR-29b, and miR-29c) [19], suggesting that blv-miR-B4-3p and the miR-29 family have similar functions. The miR-29 family is involved in cell proliferation, apoptosis, angiogenesis, and metastasis in a variety of human tumor cells [46, 99]. The blv-miR-B4-3p also promotes cell proliferation by down-regulating the expression of a transcription repressor HMG box-containing protein 1 (HBP1), which suppresses the cell cycle of ovine malignant B cell lymphoma *in vitro* [19, 22, 100]. However, the results of this study show that *HBP1* gene expression was not decreased in B cells derived from healthy BLV-infected cattle. These results suggest that BLV provirus-derived miRNAs, including blv-miR-B4-3p, modulate proliferation and apoptosis of BLV-infected B cells in an HBP1-independent manner and contribute to the increased PVL seen prior to the onset of EBL.

Of the 49 bta-miRNAs that had significant differences in their read counts between BLV-infected and uninfected cattle, 32 bta-miRNAs significantly correlated with PVL (Table 4); 31 bta-miRNAs had a negative correlation with PVL and 1 (bta-miR-375-3p) had a positive correlation with PVL. Of the 31 miRNAs that had negative correlations with PVL, 3 bta-miRNAs (bta-miR-28-5p, bta-miR-150-5p and bta-miR-197-3p) had a strong negative correlation (*r* < -0.7, *p* < 0.01). MiR-28 controls cell proliferation, is down-regulated in B-cell lymphomas [75], and reduces HTLV replication and infection [101]. The role of miR-150 in human cancer is context-dependent as this miRNA can have either oncogenic or tumor suppressor activity in cells that originate from different tissues. This is highlighted by the upregulated expression of miR-150 in B cells from chronic lymphocytic leukemia (CLL) [102, 103] but downregulated expression in chronic myeloid leukemia [104, 105] and mantle cell lymphoma [106]. MiR-197 functions as a tumor suppressor in multiple myeloma and hepatocellular carcinoma and as a key repressor of the p53-dependent apoptotic cascade in lung cancer [67, 68, 107]. Moreover, 12 of the 13 bta-miRNAs with relatively weaker negative correlation coefficients (-0.7 < r < 0.6) function as tumor suppressors and/or oncogenes. In particular, miR-146a has been deregulated in HTLV-1-transformed T-cells [108]. Taken together, these results suggest that increased PVL down-regulates the expression of bta-miRNAs, the majority of which have functions involved in suppressing cell proliferation and viral replication.

There were positive correlations between the expression of *tax/rex* mRNA and the read counts of five blv-miRNAs (blv-miR-B1-5p, blv-miR-B2-3p, blv-miR-B2-5p, blv-miR-B4-3p, and blv-miR-B5-5p). In addition, BLV *tax*/*rex* mRNA copy number showed a negative correlation with 5 bta-miRNAs (bta-miR-28-5p, bta-miR-197-3p, bta-miR-221-3p, bta-miR-22-3p, and bta-miR-151-5p), which are associated with tumorigenesis [38, 45, 48, 67, 68, 75, 76]. In HTLV infection, HTLV-1 Tax protein does not affect the expression of provirus-derived miRNA [109] whereas HTLV-1 Tax protein suppresses the expression of host genome-derived miRNAs in adult T-cell leukemia [110, 111]. Our results indicate that BLV Tax protein up-regulates the expression of provirus-derived miRNAs, such as blv-miR-B4-3p, to increase the PVL, and down-regulates some host-derived miRNA expression levels. However, the number of host-derived miRNAs that were associated with *tax/rex* mRNA was significantly reduced, and the correlation coefficient was lower than those associated with *AS1* mRNA. Therefore, the mechanisms by which Tax protein contributes to tumor development by regulating provirus-derived miRNAs differs between BLV and HTLV-1. The ability of *AS1* to reduce the expression of host-derived miRNAs might be more important than that of *tax/rex*.

Little is known about the function of the BLV *AS1* gene, which is encoded by the minus strand of BLV provirus. *AS1* transcripts are not present in the cytoplasm and AS1 protein has not been identified [20], suggesting that the *AS1* gene functions as transcripts (RNA), but not as protein. In this study, there was a positive correlation between *AS1* transcripts and the expression levels of two of the five blv-miRNAs (blv-miR-B1-5p and blv-miR-B2-5p), whereas there was a positive correlation between *tax/rex* transcripts and the read counts of the five blv-miRNAs. Furthermore, the correlation coefficient with *AS1* was lower than that with *tax/rex*. Although the reason is unknown, the interaction with bta-miRNAs might be different between *tax/rex* transcribed from the 5’ flanking region and *AS1* transcribed from the 3’ flanking region. In addition, blv-miR-B1-5p and blv-miR-B2-5p might more strongly influence *AS1* transcription than do blv-miR-B4-3p, blv-miR-B2-3p, and blv-miR-B5-5p.

Of the 31 bta-miRNAs that had a negative correlation with PVL, 24 bta-miRNAs also had a negative correlation with *AS1* mRNA expression, and the majority of the bta-miRNAs function as tumor suppressors or oncogenes (Table 4). In particular, bta-miR-150-5p, an important tumor suppresser of leukemia/lymphoma that targets Nanog (a homeobox transcription regulatory factor involved in stem cell pluripotency) [36, 112], had a strong negative correlation with *AS1* mRNA expression. MiR-150 is expressed at high levels in mature T and B cells, is downregulated in regulatory T cells (Tregs) through the action of Foxp3 [113], is downregulated in HTLV 1-infected cells, and is upregulated in adult T cell leukemia/lymphoma (ATLL) cells [114, 115]. Our data for BLV-infected B cells is consistent with the down-regulation of miR-150 in HTLV 1-infected cells. In addition, the 3’ UTR of HIV-1 mRNA is targeted by miR-150 and miR-28, and these interactions influence the ability of the virus to effectively infect CD4^+^ T cells, monocytes, and macrophages [116, 117]. MiR-150 specifically targets the signal transducer and activator of transcription 1 (STAT1) 3’ UTR, reducing STAT1 expression and dampening STAT1-dependent signaling in human T cells [118]. HTLV-I–transformed and ATL-derived cells have reduced levels of miR-150 expression, which coincides with increased STAT1 expression and STAT1-dependent signaling. STAT1 plays a role in immune modulatory functions, anti-viral responses, apoptosis, and anti-proliferative responses [119]. In addition, STAT1 can act as a potent tumor promoter of leukemia development [120]. Interestingly, HBZ interacts with STAT1 and enhances its transcriptional activities [121]. Assuming that *AS1* has the same function as HBZ, *AS1* might activate STAT1 and promote lymphomagenesis. MiR-484 and miR-1307 also function as tumor suppressors or oncogenes in several cancers [96, 97, 122, 123]. In particular, miR-484 is down-regulated in malignant B cell lymphoma [124]. Therefore, two of the three miRNAs that have strong negative correlations with *AS1* mRNA expression were associated with lymphomagenesis.

Moreover, 12 of the 15 bta-miRNAs with relatively weaker negative correlations to *AS1* mRNA expression (-0.7 < r < -0.6) also function as tumor suppressors, oncogenes, or both. Our study shows that both miR-532-5p and miR-106b-5p are down-regulated and have a negative correlation with *AS1* mRNA expression, which is consistent with the results of another study that showed that miR-532-5p and miR-106a-5p are significantly down-regulated in HTLV-1 asymptomatic carriers [125]. MiR-106b targets the cell cycle regulatory gene p21 (CDKN1A) and is also specifically downregulated in HIV-1 infected CD4^+^ T cells [126]. MiR-197 induces apoptosis and suppresses tumorigenicity in multiple myeloma [67]. MiR-221 inhibits erythroleukemic cell growth [127]. MiR-425 inhibits proliferation of CLL cells [128]. MiR-342 suppresses the proliferation and invasion of acute myeloid leukemia [51]. MiR-27a functions as a tumor suppressor gene in acute leukemia [66]. MiR-191 displays tumor-type specific roles in tumorigenesis, as miR-191 inhibits cyclin-dependent kinase 6 (CDK6) expression in thyroid follicular cancer [129]. Our results suggest that *AS1* may function to down-regulate the expression of bta-miRNAs that suppress cell proliferation, BLV replication, or both. In contrast, miR-146a is an NF-κB-dependent gene and is important in the control of Toll-like receptor and cytokine signaling [130]. In addition, miR-146a is highly expressed in HTLV-1-infected T-cell lines and is directly induced by Tax protein through the activation of NF- κB signaling [108, 131]. However, our results were inconsistent with those found in HTLV 1-infected cells. BLV *tax* and HTLV *tax* may have different functions for miR-146a. Therefore, these cellular miRNAs may also be pivotal in BLV latency and tumorigenesis.

*AS1* mRNA copy number was negatively correlated with six bta-miRNAs (bta-miR-92b-3p, bta-miR-423-3p, bta-miR-155-5p, bta-miR-26a-5p, bta-miR-532-5p, and bta-miR-107-3p), which were not associated with PVL. Five miRNAs (miR-92b, miR-423-3p, miR-155-5p, miR-26a-5p and miR-523-5p) function as both oncogene and tumor suppressor genes. In particular, miR-155- upregulation has been reported in HTLV-1 cell lines and adult T-cell leukemia (ATL) patients [114, 132]; however, our results are inconsistent with this and showed that expression levels of miR-155-5p were significantly decreased. MiR-26a-5p is frequently downregulated in various types of cancer, suggesting that these miRNAs function as tumor suppressors by targeting multiple oncogenes, whereas there are some reports that miR-26a promotes tumorigenesis [34, 133, 134]. Since sequencing reads of bta-miR-26a, as well as that of bta-miR-191-5p, were very high in B cells from both BLV-uninfected and infected cattle compared to that of other miRNAs, miR-26a seems to be necessary for B cell proliferation, survival, or both. Interestingly, the target sequence of miR26a/b exists in the 3’-UTR of Cell-Division Cycle *(CDC)6*, and *CDC6* gene expression is suppressed by miR-26a/b [135]. *CDC6* protein is a key factor for loading the helicase mini-chromosome maintenance (MCM) proteins onto replication origins for the assembly of the pre-replicative complex (pre-RC) at the M-to-G1 phase transition, which is required to establish replication licensing [136, 137]. Overexpression of *CDC6* gene has been shown to contribute to oncogenesis [138]. Therefore, it is possible that these five bta-miRNAs are affected by *AS1* specifically, as there are no associations between these bta-miRNAs and PVL. MiR-26a may be an important miRNA for BLV induced lymphomagenesis.

Three bta-miRNAs (bta-miR-363-3p, bta-miR-874-3p, and bta-miR-339b) were negatively correlated with PVL; however, these were not associated with either *tax/rex* or *AS1* mRNA copy number. Although the reason is unknown, these bta-miRNAs might be affected by PVL via other accessory genes, such as G4 or R3, rather than *tax/rex* and *AS1*.

In addition to bta-miR-375 expression significantly correlating with PVL in healthy BLV-infected cattle, at the onset of EBL, bta-miR-375 expression increased to significantly higher levels than those in healthy BLV-infected and uninfected cattle. Several organs express miR-375, which is significantly down-regulated in multiple types of cancer, although it has been found to be up-regulated in prostate and breast cancers [41]. This particular miRNA is a crucial regulator of phagocyte infiltration and the subsequent development of a tumor-promoting microenvironment [139]. In EBL, miR-375 up-regulation may be important for tumor development. Furthermore, our result has confirmed that bta-miR-375 expression levels can be used to distinguish between healthy BLV-infected and EBL cattle. This indicates that bta-miR-375 may be used as a diagnostic biomarker of EBL onset.

The deletion of the miR-15/16 cluster accelerates the proliferation of both human and mouse B cells by modulating the expression of genes so as to control cell cycle progression. In addition, the miR-15/16 cluster has been shown to be deleted or its expression down-regulated in two-thirds of B cell chronic lymphocytic leukemia (B-CLL) cases, which is characterized by the clonal expansion of CD5^+^ B cells and is similar to that seen in EBL [2, 140, 141]. In this study, however, the expression of bta-miR-16a was the most stable in B cells, among all 22 cattle, both BLV-infected and BLV-uninfected cattle, and was used as the internal control to normalize the read counts of other bta-miRNAs. Although bta-miR-16b was significantly down-regulated in BLV-infected cattle, its down-regulation was not affected by PVL or *AS1* expression. Furthermore, the expression levels of bta-miR-15a and -15b in the B cells of BLV-infected cattle did not different significantly from those in BLV-uninfected cattle. This suggests that the bta-miR-15/16 cluster may not be involved in B cell lymphoma caused by BLV.

In conclusion, our deep sequencing analysis demonstrated that BLV provirus-derived blv-miRNAs are preferentially expressed in B cells and correlate with PVL in healthy BLV-infected cattle. In contrast, the expression of some bovine-derived bta-miRNAs, which are believed to be involved in tumor and/or tumor suppression, were significantly down-regulated. These results suggest that BLV promotes lymphomagenesis by down-regulating the expression of bta-miRNAs that have tumor-suppressing functions. However, this lymphomagenesis promotion involves *AS1* and blv-miRNAs rather than the *tax/rex* genes and is associated with increased PVL. Further studies are needed to investigate the molecular function of blv-miRNAs and bta-miRNAs in the pathogenesis of EBL induced by BLV.

## Supporting information

S1 Table Bovine-derived microRNAs (bta-miRNAs) detected in B cells by deep sequencing. (XLSX)

S2 Table Comparison of bovine miRNA (bta-miRNAs) expression levels between BLV-infected and BLV-uninfected cattle. (XLSX)

S1 Fig Proviral load, BLV *tax/rex* and *AS1* mRNA expression levels in B cells derived from BLV-infected cattle. (A) Proviral load (PVL) is indicated by copies/10 ng DNA. BLV *tax/rex* and *AS1* mRNA copy numbers were normalized to *GAPDH* mRNA copy number. Data are presented as box and whisker plots, where boxes encompass values between the 5th and 95th percentiles and vertical lines represent median values. (B) There was no significant correlation between *tax/rex* and *AS1* mRNA expression (*r* = 0.2893, *p* = 0.2748). Data were analyzed by Spearman’s correlation coefficient test; *r*, correlation coefficient; *p*, *p* value.
(TIF)

S2 Fig *HBP1* mRNA expression levels in B cells derived from BLV negative and BLV-infected cattle, and an EBL derived tumor cell line, KU-17. *HBP1* mRNA copy number was normalized to *ACTB* mRNA copy number. The expression levels of *HBP1* mRNA in B cells derived from BLV-infected cattle did not differ from those of BLV-uninfected cattle (*p* = 0.3217). *HBP1* mRNA expression in the bovine B cell tumor cell line KU-17 was lower than that in B cells derived from both BLV-infected and -uninfected cattle. Data were analyzed by Kruskal-Wallis test with Steel-Dwass post-hoc test.
(TIF)

## References

[1] Stoye JP, Blomberg J, Coffin JM, Fan H, Hahn B, Neil J, et al. Family Retroviridae. In: King AM, Lefkowitz E, Adams MJ, Carstens EB, editors. Virus taxonomy: ninth report of the International Committee on Taxonomy of Viruses. 9. San Diego: Elsevier; 2011. pp. 477–495.

[2] Kettmann R, Burny A, Callebaut, Droogmans L, Mammerickx M, Willems L, et al. Bovine leukemia virus. In: Levy J, editor. The Retroviridae. 3. New York: Plenum Press; 1994. pp. 39–81.

[3] OIE. Enzootic bovine leukosis. Manual of diagnostic tests and vaccines for terrestrial Animals 2019. Paris: World Health Organization for Animal Health; 2019. pp. 1113–1124.

[4] Murakami K, Kobayashi S, Konishi M, Kameyama K-i, Tsutsui T. Nationwide Survey of Bovine Leukemia Virus Infection among Dairy and Beef Breeding Cattle in Japan from 2010–2011. J Vet Med Sci. 2013: 12–0374. 10.1292/jvms.12-0374 23563620

[5] Yang Y, Fan W, Mao Y, Yang Z, Lu G, Zhang R, et al. Bovine leukemia virus infection in cattle of China: Association with reduced milk production and increased somatic cell score. J Dairy Sci. 2016; 99: 3688–3697. doi: 10.3168/jds.2015-10580 26923050

[6] Trono KG, Pérez-Filgueira DM, Duffy S, Borca MV, Carrillo C. Seroprevalence of bovine leukemia virus in dairy cattle in Argentina: comparison of sensitivity and specificity of different detection methods. Vet Microbiol. 2001; 83: 235–248. doi: 10.1016/s0378-1135(01)00420-5 11574172

[7] VanLeeuwen JA, Tiwari A, Plaizier JC, Whiting TL. Seroprevalences of antibodies against bovine leukemia virus, bovine viral diarrhea virus, Mycobacterium avium subspecies paratuberculosis, and Neospora caninum in beef and dairy cattle in Manitoba. The Canadian Veterinary Journal. 2006; 47: 783. 16933557 PMC1524833

[8] LaDronka RM, Ainsworth S, Wilkins MJ, Norby B, Byrem TM, Bartlett PC. Prevalence of bovine leukemia virus antibodies in US dairy cattle. Vet Med Int. 2018; 2018. doi: 10.1155/2018/5831278 30534354 PMC6252197

[9] Şevik M, Avcı O, İnce ÖB. An 8-year longitudinal sero-epidemiological study of bovine leukaemia virus (BLV) infection in dairy cattle in Turkey and analysis of risk factors associated with BLV seropositivity. Trop Anim Health Prod. 2015; 47: 715–720. doi: 10.1007/s11250-015-0783-x 25708566

[10] Haghparast A, Tabatabaei Zadeh SE, Mohammadi GR. Prevalence of Bovine Leukemia Virus (BLV) antibodies in bulk tank milk of dairy cattle herds of Mashhad area, North East of Iran. J Anim Vet Adv. 2012; 11.

[11] Ochirkhuu N, Konnai S, Odbileg R, Nishimori A, Okagawa T, Murata S, et al. Detection of bovine leukemia virus and identification of its genotype in Mongolian cattle. Arch Virol. 2016; 161: 985–991. doi: 10.1007/s00705-015-2676-8 26711456

[12] Polat M, Ohno A, Takeshima S-n, Kim J, Kikuya M, Matsumoto Y, et al. Detection and molecular characterization of bovine leukemia virus in Philippine cattle. Arch Virol. 2015; 160: 285–296. doi: 10.1007/s00705-014-2280-3 25399399

[13] Hamada R, Metwally S, Polat M, Borjigin L, Ali AO, Abdel-Hady AAA, et al. Detection and Molecular Characterization of Bovine Leukemia Virus in Egyptian Dairy Cattle. Frontiers in Veterinary Science. 2020; 7. doi: 10.3389/fvets.2020.00608 33134337 PMC7511665

[14] Ndou RV, Sejesho F, Dzoma BM, Motsei LE, Nyirenda M, Bakunzi FR. A serosurvey of the prevalence of enzootic bovine leukosis in the Mafikeng area of the North West Province of South Africa. Journal of Human Ecology. 2011; 36: 53–55.

[15] Corredor-Figueroa AP, Salas S, Olaya-Galán NN, Quintero JS, Fajardo Á, Soñora M, et al. Prevalence and molecular epidemiology of bovine leukemia virus in Colombian cattle. Infect Genet Evol. 2020; 80: 104171. doi: 10.1016/j.meegid.2020.104171 31904555

[16] MAFF. Annual statistics of notifiable diseases (in Japanese): Food Safety and Consumer Bureau, Ministry of Agriculture, Forestry and Fisheries; 2019. https://www.maff.go.jp/j/syouan/douei/kansi_densen/attach/pdf/kansi_densen-165.pdf.

[17] Motoyama M, Sasaki K, Watanabe A. Wagyu and the factors contributing to its beef quality: A Japanese industry overview. Meat Science. 2016; 120: 10–18. doi: 10.1016/j.meatsci.2016.04.026 27298198

[18] Tsutsui T, Kobayashi S, Hayama Y, Yamamoto T. Fraction of bovine leukemia virus-infected dairy cattle developing enzootic bovine leukosis. Prev Vet Med. 2016; 124: 96–101. doi: 10.1016/j.prevetmed.2015.11.019 26754928

[19] Kincaid RP, Burke JM, Sullivan CS. RNA virus microRNA that mimics a B-cell oncomiR. Proc Natl Acad Sci. 2012; 109: 3077–3082. doi: 10.1073/pnas.1116107109 22308400 PMC3286953

[20] Durkin K, Rosewick N, Artesi M, Hahaut V, Griebel P, Arsic N, et al. Characterization of novel Bovine Leukemia Virus (BLV) antisense transcripts by deep sequencing reveals constitutive expression in tumors and transcriptional interaction with viral microRNAs. Retrovirology. 2016; 13: 33. doi: 10.1186/s12977-016-0267-8 27141823 PMC4855707

[21] Gillet NA, Hamaidia M, De Brogniez A, Gutierrez G, Renotte N, Reichert M, et al. Bovine leukemia virus small noncoding RNAs are functional elements that regulate replication and contribute to oncogenesis in vivo. PLoS Pathog. 2016; 12: e1005588. doi: 10.1371/journal.ppat.1005588 27123579 PMC4849745

[22] Rosewick N, Momont M, Durkin K, Takeda H, Caiment F, Cleuter Y, et al. Deep sequencing reveals abundant noncanonical retroviral microRNAs in B-cell leukemia/lymphoma. Proc Natl Acad Sci. 2013; 110: 2306–2311. doi: 10.1073/pnas.1213842110 . 23345446 PMC3568357

[23] Bartel DP. MicroRNAs: target recognition and regulatory functions. Cell. 2009; 136: 215–233. doi: 10.1016/j.cell.2009.01.002 19167326 PMC3794896

[24] Lu J, Getz G, Miska EA, Alvarez-Saavedra E, Lamb J, Peck D, et al. MicroRNA expression profiles classify human cancers. Nature. 2005; 435: 834–838. doi: 10.1038/nature03702 15944708

[25] Pfeffer S, Voinnet O. Viruses, microRNAs and cancer. Oncogene. 2006; 25: 6211. doi: 10.1038/sj.onc.1209915 17028601

[26] Suffert G, Malterer G, Hausser J, Viiliäinen J, Fender A, Contrant M, et al. Kaposi’s sarcoma herpesvirus microRNAs target caspase 3 and regulate apoptosis. PLoS Pathog. 2011; 7: e1002405. doi: 10.1371/journal.ppat.1002405 22174674 PMC3234232

[27] Koyama H, Hohdatsu T, Satake M, Kobayashi M, Ashizawa T, Sugimoto K, et al. Properties of nine continuous B-cell lines established from enzootic bovine leukosis tumors. Zentralbl Veterinarmed B. 1992; 39: 32–38. doi: 10.1111/j.1439-0450.1992.tb01134.x . 1580106

[28] Kozomara A, Birgaoanu M, Griffiths-Jones S. miRBase: from microRNA sequences to function. Nucleic Acids Res. 2019; 47: D155–D162. doi: 10.1093/nar/gky1141 30423142 PMC6323917

[29] Bai H, Shabur TMA, Kunii H, Itoh T, Kawahara M, Takahashi M. Evaluation of the immune status of peripheral blood monocytes from dairy cows during the periparturition period. Journal of Reproduction and Development. 2019; 65: 313–318. doi: 10.1262/jrd.2018-150 31061297 PMC6708849

[30] Chen P, Pan X, Zhao L, Jin L, Lin C, Quan J, et al. MicroRNA-191-5p exerts a tumor suppressive role in renal cell carcinoma. Exp Ther Med. 2018; 15: 1686–1693. doi: 10.3892/etm.2017.5581 . 29434754 PMC5774385

[31] Li H, Zhou ZQ, Yang ZR, Tong DN, Guan J, Shi BJ, et al. MicroRNA-191 acts as a tumor promoter by modulating the TET1-p53 pathway in intrahepatic cholangiocarcinoma. Hepatology. 2017; 66: 136–151. doi: 10.1002/hep.29116 . 28194813

[32] Wicik Z, Gajewska M, Majewska A, Walkiewicz D, Osińska E, Motyl T. Characterization of microRNA profile in mammary tissue of dairy and beef breed heifers. J Anim Breed Genet. 2016; 133: 31–42. doi: 10.1111/jbg.12172 26060050

[33] Miyamoto K, Seki N, Matsushita R, Yonemori M, Yoshino H, Nakagawa M, et al. Tumour-suppressive miRNA-26a-5p and miR-26b-5p inhibit cell aggressiveness by regulating PLOD2 in bladder cancer. Br J Cancer. 2016; 115: 354–363. doi: 10.1038/bjc.2016.179 27310702 PMC4973152

[34] Shen W, Song M, Liu J, Qiu G, Li T, Hu Y, et al. MiR-26a Promotes Ovarian Cancer Proliferation and Tumorigenesis. PLoS One. 2014; 9: e86871. doi: 10.1371/journal.pone.0086871 24466274 PMC3899311

[35] Jia L, Xi Q, Wang H, Zhang Z, Liu H, Cheng Y, et al. miR-142-5p regulates tumor cell PD-L1 expression and enhances anti-tumor immunity. Biochem Biophys Res Commun. 2017; 488: 425–431. doi: 10.1016/j.bbrc.2017.05.074 28511795

[36] Watanabe A, Tagawa H, Yamashita J, Teshima K, Nara M, Iwamoto K, et al. The role of microRNA-150 as a tumor suppressor in malignant lymphoma. Leukemia. 2011; 25: 1324–1334. doi: 10.1038/leu.2011.81 21502955

[37] Wang W, Zhang Y, Wang D, Gao S, Wang X, Gao H, et al. Prognostic role of microRNA-150 in various carcinomas: a meta-analysis. Onco Targets Ther. 2016: 1371. doi: 10.2147/OTT.S97969 27042106 PMC4795660

[38] Lv KT, Liu Z, Feng J, Zhao W, Hao T, Ding WY, et al. MiR-22-3p Regulates Cell Proliferation and Inhibits Cell Apoptosis through Targeting the eIF4EBP3 Gene in Human Cervical Squamous Carcinoma Cells. Int J Med Sci. 2018; 15: 142–152. doi: 10.7150/ijms.21645 . 29333098 PMC5765727

[39] Jia C-M, Tian Y-Y, Quan L-N, Jiang L, Liu A-C. miR-26b-5p suppresses proliferation and promotes apoptosis in multiple myeloma cells by targeting JAG1. Pathology—Research and Practice. 2018; 214: 1388–1394. doi: 10.1016/j.prp.2018.07.025 30098829

[40] Liu C, Zhang L, Xu R, Zheng H. miR-26b Inhibits Virus Replication Through Positively Regulating Interferon Signaling. Viral Immunol. 2018; 31: 676–682. doi: 10.1089/vim.2018.0067 . 30265587

[41] Yan JW, Lin JS, He XX. The emerging role of miR-375 in cancer. Int J Cancer. 2014; 135: 1011–1018. doi: 10.1002/ijc.28563 . 24166096

[42] Li J, Xia L, Zhou Z, Zuo Z, Xu C, Song H, et al. MiR-186-5p upregulation inhibits proliferation, metastasis and epithelial-to-mesenchymal transition of colorectal cancer cell by targeting ZEB1. Arch Biochem Biophys. 2018; 640: 53–60. doi: 10.1016/j.abb.2018.01.002 29325758

[43] Pekarsky Y, Croce CM. Role of miR-15/16 in CLL. Cell Death Differ. 2015; 22: 6–11. doi: 10.1038/cdd.2014.87 . 24971479 PMC4262785

[44] Cao J-m, Li G-z, Han M, Xu H-l, Huang K-m. MiR-30c-5p suppresses migration, invasion and epithelial to mesenchymal transition of gastric cancer via targeting MTA1. Biomed Pharmacother. 2017; 93: 554–560. doi: 10.1016/j.biopha.2017.06.084 28686969

[45] Croce CM. Causes and consequences of microRNA dysregulation in cancer. Nature reviews genetics. 2009; 10: 704–714. doi: 10.1038/nrg2634 19763153 PMC3467096

[46] Wang J-y, Zhang Q, Wang D-d, Yan W, Sha H-h, Zhao J-h, et al. MiR-29a: a potential therapeutic target and promising biomarker in tumors. Biosci Rep. 2018; 38. doi: 10.1042/BSR20171265 29217524 PMC5803495

[47] Mishan MA, Tabari MAK, Parnian J, Fallahi J, Mahrooz A, Bagheri A. Functional mechanisms of miR-192 family in cancer. Genes Chromosomes Cancer. 2020; 59: 722–735. doi: 10.1002/gcc.22889 32706406

[48] Liu C, Li W, Zhang L, Song C, Yu H. Tumor-suppressor microRNA-151-5p regulates the growth, migration and invasion of human breast cancer cells by inhibiting SCOS5. Am J Transl Res. 2019; 11: 7376–7384. . 31934285 PMC6943465

[49] Ioannidis J, Donadeu FX. Comprehensive analysis of blood cells and plasma identifies tissue-specific miRNAs as potential novel circulating biomarkers in cattle. BMC Genomics. 2018; 19: 243. doi: 10.1186/s12864-018-4646-5 29636028 PMC5894187

[50] Li X-R, Chu H-J, Lv T, Wang L, Kong S-F, Dai S-Z. miR-342-3p suppresses proliferation, migration and invasion by targeting FOXM1 in human cervical cancer. FEBS Lett. 2014; 588: 3298–3307. doi: 10.1016/j.febslet.2014.07.020 25066298

[51] Wang H, He H, Yang C. miR-342 suppresses the proliferation and invasion of acute myeloid leukemia by targeting Naa10p. Artificial Cells, Nanomedicine, and Biotechnology. 2019; 47: 3671–3676. doi: 10.1080/21691401.2019.1596930 31496296

[52] Liu S, Wang Q, Liu Y, Xia ZY. miR-425-5p suppresses tumorigenesis and DDP resistance in human-prostate cancer by targeting GSK3β and inactivating the Wnt/β-catenin signaling pathway. J Biosci. 2019; 44. . 31502580

[53] Ueda T, Volinia S, Okumura H, Shimizu M, Taccioli C, Rossi S, et al. Relation between microRNA expression and progression and prognosis of gastric cancer: a microRNA expression analysis. Lancet Oncol. 2010; 11: 136–146. doi: 10.1016/S1470-2045(09)70343-2 20022810 PMC4299826

[54] Guan G, Zhang D, Zheng Y, Wen L, Yu D, Lu Y, et al. microRNA-423-3p promotes tumor progression via modulation of AdipoR2 in laryngeal carcinoma. Int J Clin Exp Pathol. 2014; 7: 5683–5691. . 25337209 PMC4203180

[55] Sun Q, Zhao X, Liu X, Wang Y, Huang J, Jiang B, et al. miR-146a functions as a tumor suppressor in prostate cancer by targeting Rac1. Prostate. 2014; 74: 1613–1621. doi: 10.1002/pros.22878 25214035

[56] Pacifico F, Crescenzi E, Mellone S, Iannetti A, Porrino N, Liguoro D, et al. Nuclear Factor-κB Contributes to Anaplastic Thyroid Carcinomas through Up-Regulation of miR-146a. J Clin Endocrinol Metab. 2010; 95: 1421–1430. doi: 10.1210/jc.2009-1128 20061417

[57] Mansoori B, Mohammadi A, Ghasabi M, Shirjang S, Dehghan R, Montazeri V, et al. miR-142-3p as tumor suppressor miRNA in the regulation of tumorigenicity, invasion and migration of human breast cancer by targeting Bach-1 expression. J Cell Physiol. 2019; 234: 9816–9825. doi: 10.1002/jcp.27670 . 30480817

[58] Jia W, Yu T, An Q, Cao X, Pan H. MicroRNA-423-5p inhibits colon cancer growth by promoting caspase-dependent apoptosis. Exp Ther Med. 2018. doi: 10.3892/etm.2018.6288 30116373 PMC6090304

[59] Tang X, Zeng X, Huang Y, Chen S, Lin F, Yang G, et al. miR-423-5p serves as a diagnostic indicator and inhibits the proliferation and invasion of ovarian cancer. Exp Ther Med. 2018. doi: 10.3892/etm.2018.6015 29849781 PMC5960745

[60] Li S, Zhang T, Zhou X, Du Z, Chen F, Luo J, et al. The tumor suppressor role of miR-155-5p in gastric cancer. Oncol Lett. 2018. doi: 10.3892/ol.2018.8932 30008945 PMC6036547

[61] Fu X, Wen H, Jing L, Yang Y, Wang W, Liang X, et al. MicroRNA-155-5p promotes hepatocellular carcinoma progression by suppressing PTEN through the PI3K/Akt pathway. Cancer Sci. 2017; 108: 620–631. doi: 10.1111/cas.13177 28132399 PMC5406601

[62] Zhao L, Yu H, Yi S, Peng X, Su P, Xiao Z, et al. The tumor suppressor miR-138-5p targets PD-L1 in colorectal cancer. Oncotarget. 2016; 7: 45370–45384. doi: 10.18632/oncotarget.9659 27248318 PMC5216728

[63] Yang R, Liu M, Liang H, Guo S, Guo X, Yuan M, et al. miR-138-5p contributes to cell proliferation and invasion by targeting Survivin in bladder cancer cells. Mol Cancer. 2016; 15. doi: 10.1186/s12943-016-0569-4 27978829 PMC5159976

[64] Song YX, Yue ZY, Wang ZN, Xu YY, Luo Y, Xu HM, et al. MicroRNA-148b is frequently down-regulated in gastric cancer and acts as a tumor suppressor by inhibiting cell proliferation. Mol Cancer. 2011; 10: 1. doi: 10.1186/1476-4598-10-1. 21205300 PMC3024301

[65] Liu T, Tang H, Lang Y, Liu M, Li X. MicroRNA-27a functions as an oncogene in gastric adenocarcinoma by targeting prohibitin. Cancer Lett. 2009; 273: 233–242. doi: 10.1016/j.canlet.2008.08.003 . 18789835

[66] Scheibner KA, Teaboldt B, Hauer MC, Chen X, Cherukuri S, Guo Y, et al. MiR-27a Functions as a Tumor Suppressor in Acute Leukemia by Regulating 14-3-3θ. PLoS One. 2012; 7: e50895. doi: 10.1371/journal.pone.0050895 23236401 PMC3517579

[67] Yang Y, Li F, Saha MN, Abdi J, Qiu L, Chang H. miR-137 and miR-197 Induce Apoptosis and Suppress Tumorigenicity by Targeting MCL-1 in Multiple Myeloma. Clin Cancer Res. 2015; 21: 2399–2411. doi: 10.1158/1078-0432.CCR-14-1437 25724519

[68] Fiori ME, Barbini C, Haas TL, Marroncelli N, Patrizii M, Biffoni M, et al. Antitumor effect of miR-197 targeting in p53 wild-type lung cancer. Cell Death Differ. 2014; 21: 774–782. doi: 10.1038/cdd.2014.6 24488097 PMC3978312

[69] Asama H, Suzuki R, Hikichi T, Takagi T, Masamune A, Ohira H. MicroRNA let-7d targets thrombospondin-1 and inhibits the activation of human pancreatic stellate cells. Pancreatology. 2019; 19: 196–203. doi: 10.1016/j.pan.2018.10.012 30393009

[70] Li T, Ding ZL, Zheng YL, Wang W. MiR-484 promotes non-small-cell lung cancer (NSCLC) progression through inhibiting Apaf-1 associated with the suppression of apoptosis. Biomed Pharmacother. 2017; 96: 153–164. doi: 10.1016/j.biopha.2017.09.102 . 28982084

[71] Li Y, Liu Y, Yao J, Li R, Fan X. Downregulation of miR-484 is associated with poor prognosis and tumor progression of gastric cancer. Diagn Pathol. 2020; 15. doi: 10.1186/s13000-020-00946-8 32192507 PMC7082931

[72] Long M, Zhan M, Xu S, Yang R, Chen W, Zhang S, et al. miR-92b-3p acts as a tumor suppressor by targeting Gabra3 in pancreatic cancer. Mol Cancer. 2017; 16. doi: 10.1186/s12943-017-0723-7 29078789 PMC5659029

[73] Wang C, Uemura M, Tomiyama E, Matsushita M, Koh Y, Nakano K, et al. MicroRNA‐92b‐3p is a prognostic oncomiR that targets TSC1 in clear cell renal cell carcinoma. Cancer Sci. 2020; 111: 1146–1155. doi: 10.1111/cas.14325 31975504 PMC7156823

[74] Liu D, Tao T, Xu B, Chen S, Liu C, Zhang L, et al. MiR-361-5p acts as a tumor suppressor in prostate cancer by targeting signal transducer and activator of transcription-6(STAT6). Biochem Biophys Res Commun. 2014; 445: 151–156. doi: 10.1016/j.bbrc.2014.01.140 . 24491557

[75] Schneider C, Setty M, Holmes AB, Maute RL, Leslie CS, Mussolin L, et al. MicroRNA 28 controls cell proliferation and is down-regulated in B-cell lymphomas. Proc Natl Acad Sci. 2014; 111: 8185–8190. doi: 10.1073/pnas.1322466111 . 24843176 PMC4050621

[76] Li L, Zhu X, Shou T, Yang L, Cheng X, Wang J, et al. MicroRNA-28 promotes cell proliferation and invasion in gastric cancer via the PTEN/PI3K/AKT signalling pathway. Mol Med Rep. 2018; 17: 4003–4010. doi: 10.3892/mmr.2017.8299 . 29257342

[77] Li X, Liu X, Xu W, Zhou P, Gao P, Jiang S, et al. c-MYC-regulated miR-23a/24-2/27a cluster promotes mammary carcinoma cell invasion and hepatic metastasis by targeting Sprouty2. J Biol Chem. 2013; 288: 18121–18133. doi: 10.1074/jbc.M113.478560 . 23649631 PMC3689956

[78] Wang G, Li B, Fu Y, He M, Wang J, Shen P, et al. miR-23a suppresses proliferation of osteosarcoma cells by targeting SATB1. Tumour Biol. 2015; 36: 4715–4721. doi: 10.1007/s13277-015-3120-0 . 25619478

[79] Ivanovska I, Ball AS, Diaz RL, Magnus JF, Kibukawa M, Schelter JM, et al. MicroRNAs in the miR-106b family regulate p21/CDKN1A and promote cell cycle progression. Mol Cell Biol. 2008; 28: 2167–2174. doi: 10.1128/MCB.01977-07 . 18212054 PMC2268421

[80] Fang L, Sørensen P, Sahana G, Panitz F, Su G, Zhang S, et al. MicroRNA-guided prioritization of genome-wide association signals reveals the importance of microRNA-target gene networks for complex traits in cattle. Sci Rep. 2018; 8. doi: 10.1038/s41598-018-27729-y 29921979 PMC6008395

[81] Jiang Z, Guo J, Xiao B, Miao Y, Huang R, Li D, et al. Increased expression of miR-421 in human gastric carcinoma and its clinical association. J Gastroenterol. 2010; 45: 17–23. doi: 10.1007/s00535-009-0135-6 . 19802518

[82] Xue L, Yang D. MiR-421 inhibited proliferation and metastasis of colorectal cancer by targeting MTA1. J BUON. 2018; 23: 1633–1639. . 30610787

[83] Huang L, Tang X, Shi X, Su L. miR-532-5p promotes breast cancer proliferation and migration by targeting RERG. Exp Ther Med. 2019. doi: 10.3892/etm.2019.8186 31853317 PMC6909632

[84] Zhai W, Ma J, Zhu R, Xu C, Zhang J, Chen Y, et al. MiR-532-5p suppresses renal cancer cell proliferation by disrupting the ETS1-mediated positive feedback loop with the KRAS-NAP1L1/P-ERK axis. Br J Cancer. 2018; 119: 591–604. doi: 10.1038/s41416-018-0196-5 30082686 PMC6162242

[85] Dong J, Geng J, Tan W. MiR-363-3p suppresses tumor growth and metastasis of colorectal cancer via targeting SphK2. Biomed Pharmacother. 2018; 105: 922–931. doi: 10.1016/j.biopha.2018.06.052 . 30021386

[86] Li Z, Wang H, Chen L, Wang L, Liu X, Ru C, et al. Identification and characterization of novel and differentially expressed microRNAs in peripheral blood from healthy and mastitis Holstein cattle by deep sequencing. Anim Genet. 2014; 45: 20–27. doi: 10.1111/age.12096 24308606

[87] Hu S, Ran Y, Chen W, Zhang Y, Xu Y. MicroRNA-326 inhibits cell proliferation and invasion, activating apoptosis in hepatocellular carcinoma by directly targeting LIM and SH3 protein 1. Oncol Rep. 2017; 38: 1569–1578. doi: 10.3892/or.2017.5810 . 28713953

[88] Zhou J, Xu T, Yan Y, Qin R, Wang H, Zhang X, et al. MicroRNA-326 functions as a tumor suppressor in glioma by targeting the Nin one binding protein (NOB1). PLoS One. 2013; 8: e68469. doi: 10.1371/journal.pone.0068469. 23869222 PMC3711818

[89] Xia H, Long J, Zhang R, Yang X, Ma Z. MiR-32 contributed to cell proliferation of human breast cancer cells by suppressing of PHLPP2 expression. Biomed Pharmacother. 2015; 75: 105–110. doi: 10.1016/j.biopha.2015.07.037 . 26276160

[90] Zhang Z, Lei B, Wu H, Zhang X, Zheng N. Tumor suppressive role of miR-194-5p in glioblastoma multiforme. Mol Med Rep. 2017; 16: 9317–9322. doi: 10.3892/mmr.2017.7826 . 29152664 PMC5779985

[91] Li W, Wang S, He H, Qin J, Cheng X, Zhao H, et al. Expression and function of Ndel1 during the differentiation of neural stem cells induced by hippocampal exosomesticle. Stem Cell Res Ther. 2021; 12. doi: 10.1186/s13287-020-02119-2 33422130 PMC7796549

[92] Zhang X, Tang J, Zhi X, Xie K, Wang W, Li Z, et al. miR-874 functions as a tumor suppressor by inhibiting angiogenesis through STAT3/VEGF-A pathway in gastric cancer. Oncotarget. 2015; 6: 1605–1617. doi: 10.18632/oncotarget.2748 25596740 PMC4359318

[93] Son D, Kim Y, Lim S, Kang HG, Kim DH, Park JW, et al. miR-374a-5p promotes tumor progression by targeting ARRB1 in triple negative breast cancer. Cancer Lett. 2019; 454: 224–233. doi: 10.1016/j.canlet.2019.04.006 . 31004703

[94] Chen Y, Jiang J, Zhao M, Luo X, Liang Z, Zhen Y, et al. microRNA-374a suppresses colon cancer progression by directly reducing CCND1 to inactivate the PI3K/AKT pathway. Oncotarget. 2106; 7: 41306–41319. doi: 10.18632/oncotarget.9320 27191497 PMC5173061

[95] Do DN, Li R, Dudemaine P-L, Ibeagha-Awemu EM. MicroRNA roles in signalling during lactation: an insight from differential expression, time course and pathway analyses of deep sequence data. Sci Rep. 2017; 7: 44605. doi: 10.1038/srep44605 28317898 PMC5357959

[96] Chen S, Wang L, Yao B, Liu Q, Guo C. miR-1307-3p promotes tumor growth and metastasis of hepatocellular carcinoma by repressing DAB2 interacting protein. Biomed Pharmacother. 2019; 117: 109055. doi: 10.1016/j.biopha.2019.109055. 31176165

[97] Zheng Y, Zheng Y, Lei W, Xiang L, Chen M. miR-1307-3p overexpression inhibits cell proliferation and promotes cell apoptosis by targeting ISM1 in colon cancer. Mol Cell Probes. 2019; 48: 101445. doi: 10.1016/j.mcp.2019.101445. 31513891

[98] Kobayashi T, Inagaki Y, Ohnuki N, Sato R, Murakami S, Imakawa K. Increasing Bovine leukemia virus (BLV) proviral load is a risk factor for progression of Enzootic bovine leucosis: A prospective study in Japan. Prev Vet Med. 2019. doi: 10.1016/j.prevetmed.2019.04.009. 31079891

[99] Jiang H, Zhang G, Wu J-H, Jiang C-P. Diverse roles of miR-29 in cancer. Oncol Rep. 2014; 31: 1509–1516. doi: 10.3892/or.2014.3036 24573597

[100] Bollaert E, de Rocca Serra A, Demoulin J-B. The HMG box transcription factor HBP1: a cell cycle inhibitor at the crossroads of cancer signaling pathways. Cell Mol Life Sci. 2019; 76: 1529–1539. doi: 10.1007/s00018-019-03012-9 30683982 PMC11105191

[101] Bai XT, Nicot C. miR-28-3p is a cellular restriction factor that inhibits human T cell leukemia virus, type 1 (HTLV-1) replication and virus infection. J Biol Chem. 2015; 290: 5381–5390. doi: 10.1074/jbc.M114.626325 . 25568327 PMC4342455

[102] Papakonstantinou N, Ntoufa S, Chartomatsidou E, Papadopoulos G, Hatzigeorgiou A, Anagnostopoulos A, et al. Differential microRNA Profiles and Their Functional Implications in Different Immunogenetic Subsets of Chronic Lymphocytic Leukemia. Mol Med. 2013; 19: 115–123. doi: 10.2119/molmed.2013.00005 23615967 PMC3667214

[103] Mraz M, Chen L, Rassenti LZ, Ghia EM, Li H, Jepsen K, et al. miR-150 influences B-cell receptor signaling in chronic lymphocytic leukemia by regulating expression of GAB1 and FOXP1. Blood. 2014; 124: 84–95. doi: 10.1182/blood-2013-09-527234 24787006 PMC4125356

[104] Morris VA, Zhang A, Yang T, Stirewalt DL, Ramamurthy R, Meshinchi S, et al. MicroRNA-150 Expression Induces Myeloid Differentiation of Human Acute Leukemia Cells and Normal Hematopoietic Progenitors. PLoS One. 2013; 8: e75815. doi: 10.1371/journal.pone.0075815 24086639 PMC3782459

[105] Machová Poláková K, Lopotová T, Klamová H, Burda P, Trněný M, Stopka T, et al. Expression patterns of microRNAs associated with CML phases and their disease related targets. Mol Cancer. 2011; 10: 41. doi: 10.1186/1476-4598-10-41 21501493 PMC3102634

[106] Zhao J-J, Lin J, Lwin T, Yang H, Guo J, Kong W, et al. microRNA expression profile and identification of miR-29 as a prognostic marker and pathogenetic factor by targeting CDK6 in mantle cell lymphoma. Blood. 2010; 115: 2630–2639. doi: 10.1182/blood-2009-09-243147 20086245 PMC2852365

[107] Ni JS, Zheng H, Huang ZP, Hong YG, Ou YL, Tao YP, et al. MicroRNA-197-3p acts as a prognostic marker and inhibits cell invasion in hepatocellular carcinoma. Oncol Lett. 2018. doi: 10.3892/ol.2018.9848 30675297 PMC6341871

[108] Pichler K, Schneider G, Grassmann R. MicroRNA miR-146a and further oncogenesis-related cellular microRNAs are dysregulated in HTLV-1-transformed T lymphocytes. Retrovirology. 2008; 5: 100. doi: 10.1186/1742-4690-5-100 19014482 PMC2628945

[109] Ruggero K, Corradin A, Zanovello P, Amadori A, Bronte V, Ciminale V, et al. Role of microRNAs in HTLV-1 infection and transformation. Mol Aspects Med. 2010; 31: 367–382. doi: 10.1016/j.mam.2010.05.001 20600265

[110] Rahman S, Quann K, Pandya D, Singh S, Khan ZK, Jain P. HTLV-1 Tax mediated downregulation of miRNAs associated with chromatin remodeling factors in T cells with stably integrated viral promoter. PLoS One. 2012; 7. 10.1371/journal.pone.0034490 PMC3319589 22496815

[111] Yamagishi M, Nakano K, Miyake A, Yamochi T, Kagami Y, Tsutsumi A, et al. Polycomb-mediated loss of miR-31 activates NIK-dependent NF-κB pathway in adult T cell leukemia and other cancers. Cancer Cell. 2012; 21: 121–135. doi: 10.1016/j.ccr.2011.12.015 22264793

[112] Xu D-d, Zhou P-j, Wang Y, Zhang Y, Zhang R, Zhang L, et al. miR-150 suppresses the proliferation and tumorigenicity of leukemia stem cells by targeting the nanog signaling pathway. Front Pharmacol. 2016; 7: 439. doi: 10.3389/fphar.2016.00439 27917123 PMC5114241

[113] Cobb BS, Hertweck A, Smith J, O’Connor E, Graf D, Cook T, et al. A role for Dicer in immune regulation. J Exp Med. 2006; 203: 2519–2527. doi: 10.1084/jem.20061692 17060477 PMC2118134

[114] Bellon M, Lepelletier Y, Hermine O, Nicot C. Deregulation of microRNA involved in hematopoiesis and the immune response in HTLV-I adult T-cell leukemia. Blood. 2009; 113: 4914–4917. doi: 10.1182/blood-2008-11-189845 19246560 PMC2686141

[115] Yeung ML, Yasunaga J-i, Bennasser Y, Dusetti N, Harris D, Ahmad N, et al. Roles for microRNAs, miR-93 and miR-130b, and tumor protein 53–induced nuclear protein 1 tumor suppressor in cell growth dysregulation by human T-cell lymphotrophic virus 1. Cancer Res. 2008; 68: 8976–8985. doi: 10.1158/0008-5472.CAN-08-0769 18974142 PMC2596768

[116] Wang X, Ye L, Hou W, Zhou Y, Wang Y-J, Metzger DS, et al. Cellular microRNA expression correlates with susceptibility of monocytes/macrophages to HIV-1 infection. Blood. 2009; 113: 671–674. doi: 10.1182/blood-2008-09-175000 19015395 PMC2628373

[117] Huang J, Wang F, Argyris E, Chen K, Liang Z, Tian H, et al. Cellular microRNAs contribute to HIV-1 latency in resting primary CD4+ T lymphocytes. Nat Med. 2007; 13: 1241–1247. doi: 10.1038/nm1639 . 17906637

[118] Moles R, Bellon M, Nicot C. STAT1: A Novel Target of miR-150 and miR-223 Is Involved in the Proliferation of HTLV-I–Transformed and ATL Cells. Neoplasia. 2015; 17: 449–462. doi: 10.1016/j.neo.2015.04.005 26025667 PMC4468372

[119] Decker T, Stockinger S, Karaghiosoff M, Müller M, Kovarik P. IFNs and STATs in innate immunity to microorganisms. J Clin Invest. 2002; 109: 1271–1277. doi: 10.1172/JCI15770 12021240 PMC150987

[120] Kovacic B, Stoiber D, Moriggl R, Weisz E, Ott RG, Kreibich R, et al. STAT1 acts as a tumor promoter for leukemia development. Cancer Cell. 2006; 10: 77–87. doi: 10.1016/j.ccr.2006.05.025 16843267

[121] Higuchi Y, Yasunaga J-i, Mitagami Y, Ohshima K, Matsuoka M. HTLV-1 Dysregulates IL-6 and IL-10-JAK/STAT Signaling and Induces Leukemia/Lymphoma of Mature CD4+ T Cells with Regulatory T-Cell-like Signatures: American Society of Hematology Washington, DC; 2019.

[122] Mei Q, Xue G, Li X, Wu Z, Li X, Yan H, et al. Methylation-induced loss of miR-484 in microsatellite-unstable colorectal cancer promotes both viability and IL-8 production via CD137L. The Journal of Pathology. 2015; 236: 165–174. doi: 10.1002/path.4525 25727216

[123] Merhautova J, Hezova R, Poprach A, Kovarikova A, Radova L, Svoboda M, et al. miR-155 and miR-484 are associated with time to progression in metastatic renal cell carcinoma treated with sunitinib. BioMed research international. 2015; 2015. doi: 10.1155/2015/941980 26064968 PMC4433647

[124] Ferrer G, Navarro A, Hodgson K, Aymerich M, Pereira A, Baumann T, et al. MicroRNA expression in chronic lymphocytic leukemia developing autoimmune hemolytic anemia. Leuk Lymphoma. 2013; 54: 2016–2022. doi: 10.3109/10428194.2012.763123 . 23286334

[125] Valadao De Souza DR, Pessoa R, Nacimento A, Nukui Y, Pereira J, Casseb J, et al. Small RNA profiles of HTLV-1 asymptomatic carriers with monoclonal and polyclonal rearrangement of the T-cell antigen receptor γ-chain using massively parallel sequencing: A pilot study. Oncol Lett. 2020. doi: 10.3892/ol.2020.11803 32782548 PMC7400997

[126] Guha D, Mancini A, Sparks J, Ayyavoo V. HIV-1 Infection Dysregulates Cell Cycle Regulatory Protein p21 in CD4+ T Cells through miR-20a and miR-106b Regulation. J Cell Biochem. 2016: n/a-n/a. doi: 10.1002/jcb.25489 26755399

[127] Felli N, Fontana L, Pelosi E, Botta R, Bonci D, Facchiano F, et al. MicroRNAs 221 and 222 inhibit normal erythropoiesis and erythroleukemic cell growth via kit receptor down-modulation. Proc Natl Acad Sci. 2005; 102: 18081–18086. doi: 10.1073/pnas.0506216102 16330772 PMC1312381

[128] Chen J, Li Y, Xie X. MicroRNA-425 inhibits proliferation of chronic lymphocytic leukaemia cells through regulation of the Bruton’s tyrosine kinase/phospholipase Cγ2 signalling pathway. Exp Ther Med. 2020; 20: 1169–1175. doi: 10.3892/etm.2020.8771 32742355 PMC7388289

[129] Colamaio M, Borbone E, Russo L, Bianco M, Federico A, Califano D, et al. miR-191 Down-Regulation Plays a Role in Thyroid Follicular Tumors through CDK6 Targeting. The Journal of Clinical Endocrinology & Metabolism. 2011; 96: E1915–E1924. doi: 10.1210/jc.2011-0408 21956418

[130] Taganov KD, Boldin MP, Chang KJ, Baltimore D. NF- B-dependent induction of microRNA miR-146, an inhibitor targeted to signaling proteins of innate immune responses. Proc Natl Acad Sci. 2006; 103: 12481–12486. doi: 10.1073/pnas.0605298103 16885212 PMC1567904

[131] Tomita M, Tanaka Y, Mori N. MicroRNA miR-146a is induced by HTLV-1 tax and increases the growth of HTLV-1-infected T-cells. Int J Cancer. 2012; 130: 2300–2309. doi: 10.1002/ijc.25115 20017139

[132] Tomita M. Important roles of cellular microRNA miR-155 in leukemogenesis by human T-cell leukemia virus type 1 infection. International Scholarly Research Notices. 2012; 2012. doi: 10.5402/2012/978607 23762762 PMC3671690

[133] Gao J, Liu Q-G. The role of miR-26 in tumors and normal tissues (Review). Oncol Lett. 2011; 2: 1019–1023. doi: 10.3892/ol.2011.413 22848262 PMC3406571

[134] Fukumoto I, Hanazawa T, Kinoshita T, Kikkawa N, Koshizuka K, Goto Y, et al. MicroRNA expression signature of oral squamous cell carcinoma: functional role of microRNA-26a/b in the modulation of novel cancer pathways. Br J Cancer. 2015; 112: 891–900. doi: 10.1038/bjc.2015.19 25668004 PMC4453953

[135] Zhang X, Xiao D, Wang Z, Zou Y, Huang L, Lin W, et al. MicroRNA-26a/b Regulate DNA Replication Licensing, Tumorigenesis, and Prognosis by Targeting CDC6 in Lung Cancer. Mol Cancer Res. 2014; 12: 1535–1546. doi: 10.1158/1541-7786.MCR-13-0641 25100863

[136] Sclafani RA, Holzen TM. Cell Cycle Regulation of DNA Replication. Annu Rev Genet. 2007; 41: 237–280. doi: 10.1146/annurev.genet.41.110306.130308 17630848 PMC2292467

[137] Nishitani H, Lygerou Z. Control of DNA replication licensing in a cell cycle. Genes Cells. 2002; 7: 523–534. doi: 10.1046/j.1365-2443.2002.00544.x 12059957

[138] Borlado LR, Méndez J. CDC6: from DNA replication to cell cycle checkpoints and oncogenesis. Carcinogenesis. 2008; 29: 237–243. doi: 10.1093/carcin/bgm268 18048387

[139] Frank AC, Ebersberger S, Fink AF, Lampe S, Weigert A, Schmid T, et al. Apoptotic tumor cell-derived microRNA-375 uses CD36 to alter the tumor-associated macrophage phenotype. Nat Commun. 2019; 10: 1135. doi: 10.1038/s41467-019-08989-2. 30850595 PMC6408494

[140] Calin GA, Dumitru CD, Shimizu M, Bichi R, Zupo S, Noch E, et al. Frequent deletions and down-regulation of micro- RNA genes miR15 and miR16 at 13q14 in chronic lymphocytic leukemia. Proc Natl Acad Sci. 2002; 99: 15524–15529. doi: 10.1073/pnas.242606799 . 12434020 PMC137750

[141] Klein U, Lia M, Crespo M, Siegel R, Shen Q, Mo T, et al. The DLEU2/miR-15a/16-1 Cluster Controls B Cell Proliferation and Its Deletion Leads to Chronic Lymphocytic Leukemia. 2010; 17: 28–40. doi: 10.1016/j.ccr.2009.11.019 20060366

