# Supplementary figures and images for: Characterization of microRNA expression in B cells derived from Japanese black cattle naturally infected with bovine leukemia virus by deep sequencing
Source: PLoS One. 2021 Sep 10;16(9):e0256588. doi: 10.1371/journal.pone.0256588 (PMC8432782; doi:10.1371/journal.pone.0256588)

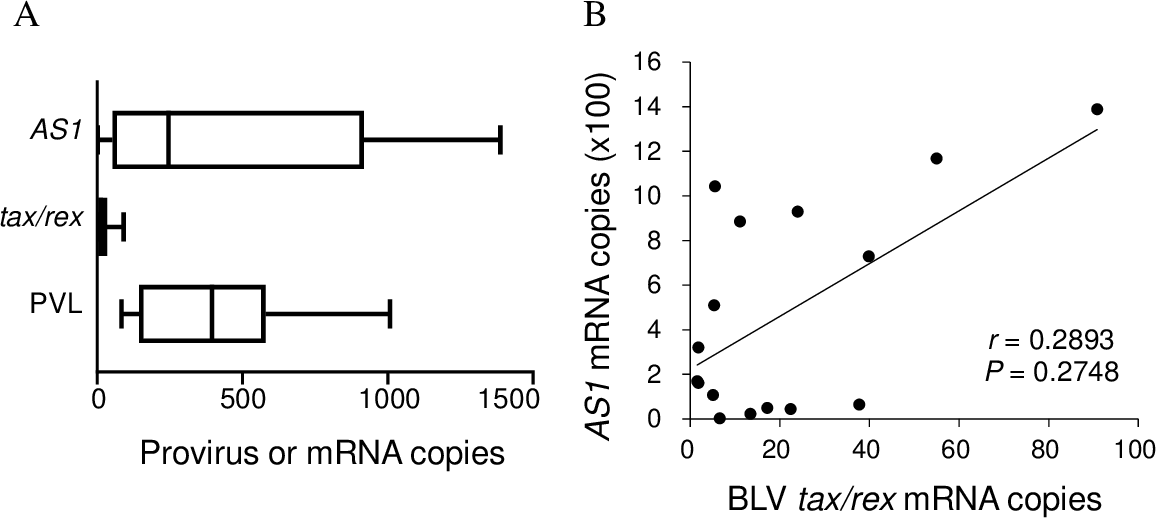

Supplement: S1 Fig — (A) Proviral load (PVL) is indicated by copies/10 ng DNA. BLV tax/rex and AS1 mRNA copy numbers were normalized to GAPDH mRNA copy number. Data are presented as box and whisker plots, where boxes encompass values between the 5th and 95th percentiles and vertical lines represent median values. (B) There was no significant correlation between tax/rex and AS1 mRNA expression (r = 0.2893, p = 0.2748). Data were analyzed by Spearman’s correlation coefficient test; r, correlation coefficient; p, p value. (TIF) [file pone.0256588.s003.tif]

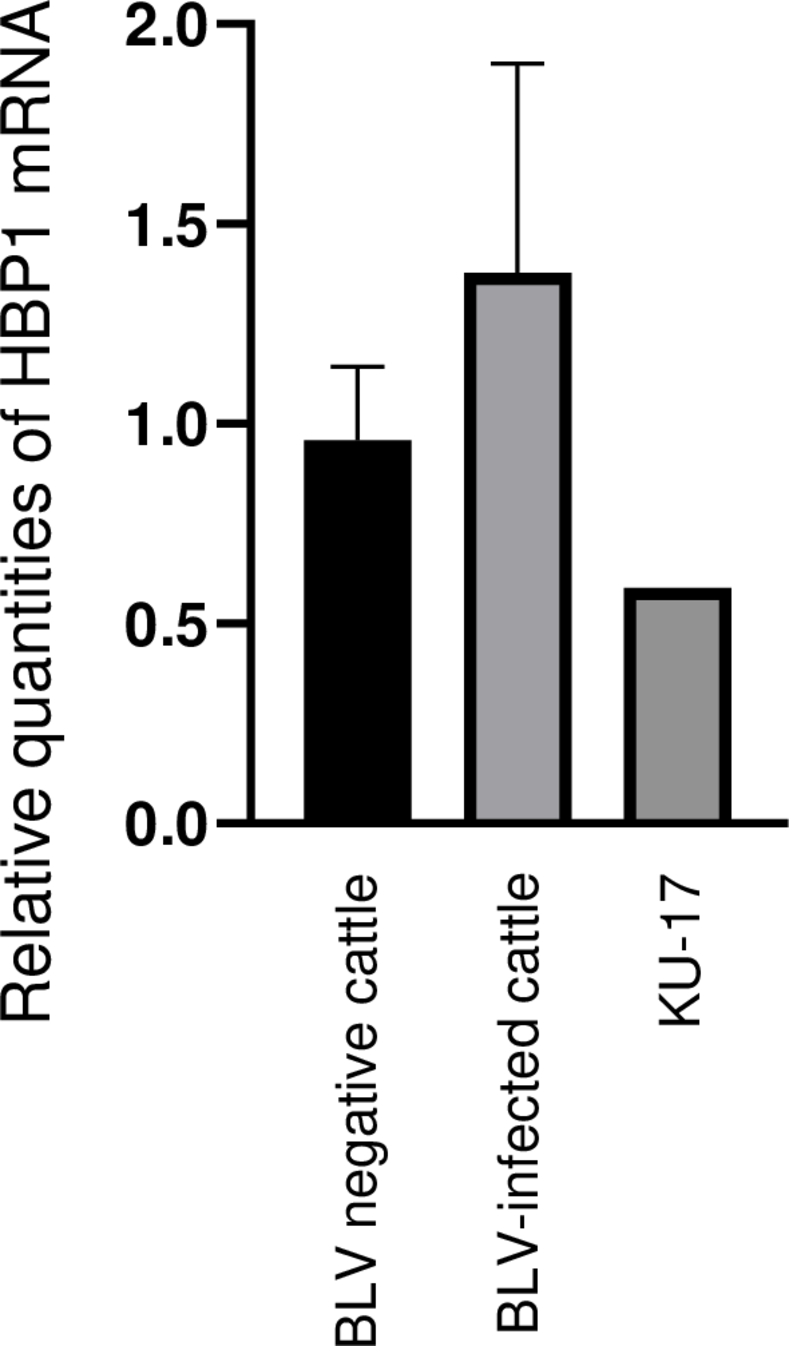

Supplement: S2 Fig — HBP1 mRNA copy number was normalized to ACTB mRNA copy number. The expression levels of HBP1 mRNA in B cells derived from BLV-infected cattle did not differ from those of BLV-uninfected cattle (p = 0.3217). HBP1 mRNA expression in the bovine B cell tumor cell line KU-17 was lower than that in B cells derived from both BLV-infected and -uninfected cattle. Data were analyzed by Kruskal-Wallis test with Steel-Dwass post-hoc test. (TIF) [file pone.0256588.s004.tif]
