# Supplementary material for: Characterization of microRNA expression in B cells derived from Japanese black cattle naturally infected with bovine leukemia virus by deep sequencing
Source: PLoS One. 2021 Sep 10;16(9):e0256588. doi: 10.1371/journal.pone.0256588 (PMC8432782; doi:10.1371/journal.pone.0256588)
Supplement: Attachment — Submitted filename: Revision note_210714.docx [file pone.0256588.s006.docx]

PONE-D-20-35708R2

Characterization of microRNA expression in B cells derived from Japanese black cattle naturally infected with bovine leukemia virus by deep sequencing

**Reviewer #1:**

**I would like to thank the authors for taking the time to address my recommendations. However, I still have some concerns regarding the presentation and analysis of the sequencing results.**

**1. Sequencing data**

**At the moment, the bioinformatics are vague by the way they are presented.**

**Lines 200-205: sequencing reads were processed with CLC Genomics Workbench software (Ver. 9.5.5; Qiagen KK) to obtain the final miRNA counts present in each sample. Briefly, adapter sequences were removed from sequencing reads and the remaining sequences were compared against the bovine miRNA database in miRBase 22 [28] (http://www.mirbase.org/blog/2018/03/mirbase-22-release/) with CLC software for miRNA gene identification, annotation, and quantification.**

We modified the sentences as below according reviewer’s suggestion. Please read revised manuscript.

Lines 200-218: sequencing reads were processed with CLC Genomics Workbench software (Ver. 9.5.5; Qiagen KK) to obtain the final miRNA counts present in each sample (see Qiagen tutorial manual for small RNA Analysis using Illumina Data for detail; https://resources.qiagenbioinformatics.com/tutorials/Small_RNA_analysis_Illumina.pdf). Briefly, adapter sequences were removed from the partial adapter sequences of the fastq file. Adapter trimming parameter was as they are set by default, i.e., mismatch cost and gap cost were 2 and 3, respectively and match threshold was selected to “allow end matches”, and the minimum score at end was set to 6. Subsequently, for sequence filtering, the minimum and maximum length values were used as default values, i.e., reads were discarded below length 15 and above length 55, and sample threshold was set to 1 as minimum sampling count. The number of copies of each of the resulting small RNAs was counted. To annotate the small RNA sample, the bovine miRNA database in miRBase 22 [28] (<http://www.mirbase.org/blog/2018/03/mirbase-22-release/>) was downloaded. The trimmed sequences were compared against the bovine miRNA database with CLC software for miRNA gene identification, annotation, and quantification. Specify match parameters were used as default values, i.e., mature length variants (IsomiRs) were set to additional upstream bases; 2, additional downstream bases; 2, missing upstream bases; 2 and missing downstream bases; 2. Alignment setting was set to maximum mismatches 2.

**Line 283 and Supplementary table 1: In the text is mentioned that "560 bovine-derived miRNAs (bta-miRNAs) were detected out of 1,064 currently registered in the database (miRBase) (S1 Table)." In S1 table there are 614 miRNAs.**

**In my opinion, since this study is heavily based on the sequencing results, there should be more data regarding the sequencing data and data analysis not only in the S1 table but also in text.**

**For example, in S1 table there are only 3 columns with the "No.", "bta-miRNA name" and "No. of animals with miRNA detected". I think that it would be useful for the reader to have all the sequencing details, such as reads from each miRNA for each animal.**

Thank you for pointing out the numbers that were left uncorrected. We corrected the number 560 to 614 in line 297.

We made new S1 table according to reviewer’s suggestion.

**At the moment it is not clear why you focused on the 49 miRNAs mentioned in Table 3 out of the total 614 mentioned in S1.**

Although we mentioned about the reason why we focused on the 49 miRNAs in line 300-301 of previous revised manuscript R1, the sentence has been modified to make it clearer as follows in line 314-317 of revised manuscript R2.

We focused on 49 bta-miRNAs because these miRNAs showed significant differences between BLV-infected and uninfected cattle. Among 49 bta-miRNAs, 48 bta-miRNAs in BLV-infected cattle were significantly decreased compared to those in uninfected cattle (*p* < 0.05, Table 3).

**Did you normalise the reads somehow? For example, reads per million mapped? Did you calculate a false discovery rate? How did you do the differential expression analysis between BLV +ve and BLV -ve?**

The read counts were normalized using bta-miR-16a-5p reads. We described it in line 298-299 of previous revised manuscript R1 and in line 312 of revised manuscript R2.

In the revised manuscript, the distributions of the present continuous variables were all reviewed. We then re-analyzed each of them using a non-parametric approach because the data were not normally distributed. For the continuous variables, such as expression of BLV miRNAs, if an arbitrary miRNA had two levels of origin, such as BLV-infected and BLV-uninfected cattle, we performed a Mann-Whitney test. In the case of three levels of origin, such as BLV-negative, BLV-positive, and EBL cattle, we used the Kruskal-Wallis test first, and if the result was significant (p < 0.05), we performed a Steel-Dwass post-hoc test in consideration of false discovery rates. Through these analyses, we found that some results differed from the first (original) manuscript. Finally, we prepared the revised manuscript R1, reflecting all the results obtained from the non-parametric approach.

Although these issues have already been mentioned in lines 266–275 of the revised manuscript R1, we welcome any alternative suggestions to describe these details that would be more easily understood.

**2. Suppl table 2**

**Here, you are presenting the blv-miRNA read counts of the BLV +ve animals. You are only presenting the 16 that are BLV +ve EBL -ve. Why did you exclude the 5 that are BLV +ve EBL +ve?**

We have only five EBL samples so far and we have not analyzed the miRNAs of BLV-positive and EBL-positive samples by using microRNA deep sequencing. We would like to do the miRNAs deep sequencing analysis after obtaining sufficient number of samples in near future. Thank you very much.

Also, only bta-miR-375 expression in EBL samples have been analyzed by using quantitative RT-PCR, not by using microRNA deep sequencing (Fig. 2H). To clear this point, we have revised the sentences as follows.

Line 349-352:

When bta-miR-375 expression was compared among BLV-uninfected, BLV-infected, and cattle with EBL via quantitative RT-PCR, the levels were significantly higher in EBL cattle than in BLV-uninfected and BLV-infected cattle (BLV-uninfected vs EBL, *p* = 0.0096; BLV-infected vs EBL, *p* = 0.0245) (Fig. 2H).

Line 368-370:

(H) Levels of bta-miR-375 expression measured by quantitative RT-PCR in B cells derived from BLV-uninfected (n = 8) and BLV-infected (n = 5) cattle, and in B cell lymphomas (n = 5) derived from EBL cattle.

**3. Suppl 3**

**I would suggest that you add a caption describing the figure without the reader to have to go to text to find out the details. I would include the stat results, such as p-value, etc. The same goes for Suppl 4.**

We have modified the caption of S3 Fig according to reviewer’s suggestion.

**4. Suppl 4**

**The caption says S6. Please amend as suppl 3.**

We have modified the caption of S4 Fig according to reviewer’s suggestion.

**Reviewer 2 - The first (and most important) point made by this reviewer has not been addressed, i.e. differential gene expression results obtained by sequencing have not been corrected for Type I error, e.g. using FDR or other suitable method. As Reviewer 2 pointed out, without this correction it is not possible to determine the reliability of the significant comparisons presented as false positives have not been excluded from the list of significant miRNAs.**

In the revised manuscript, the distributions of the present continuous variables were all reviewed. We then re-analyzed each of them using a non-parametric approach because the data were not normally distributed. For the continuous variables, such as expression of BLV miRNAs, if an arbitrary miRNA had two levels of origin, such as BLV-infected and BLV-uninfected cattle, we performed a Mann-Whitney test. In the case of three levels of origin, such as BLV-negative, BLV-positive, and EBL cattle, we used the Kruskal-Wallis test first, and if the result was significant (p < 0.05), we performed a Steel-Dwass post-hoc test in consideration of false discovery rates. Through these analyses, we found that some results differed from the first (original) version of the manuscript. Finally, we prepared the revised manuscript R1, reflecting all the results obtained from the non-parametric approach.

Although these issues have already been mentioned in lines 266–275 of the revised manuscript R1, we welcome any alternative suggestions to describe these details that would be more easily understood.
